# A Dynamic Model of Post-Traumatic Stress Disorder for Military Personnel and Veterans

**DOI:** 10.1371/journal.pone.0161405

**Published:** 2016-10-07

**Authors:** Navid Ghaffarzadegan, Alireza Ebrahimvandi, Mohammad S. Jalali

**Affiliations:** 1 Industrial and Systems Engineering, Virginia Tech, Blacksburg, Virginia, United States of America; 2 Sloan School of Management, Massachusetts Institute of Technology, Cambridge, Massachusetts, United States of America; City of Hope, UNITED STATES

## Abstract

Post-traumatic stress disorder (PTSD) stands out as a major mental illness; however, little is known about effective policies for mitigating the problem. The importance and complexity of PTSD raise critical questions: What are the trends in the population of PTSD patients among military personnel and veterans in the postwar era? What policies can help mitigate PTSD? To address these questions, we developed a system dynamics simulation model of the population of military personnel and veterans affected by PTSD. The model includes both military personnel and veterans in a “system of systems.” This is a novel aspect of our model, since many policies implemented at the military level will potentially influence (and may have side effects on) veterans and the Department of Veterans Affairs. The model is first validated by replicating the historical data on PTSD prevalence among military personnel and veterans from 2000 to 2014 (datasets from the Department of Defense, the Institute of Medicine, the Department of Veterans Affairs, and other sources). The model is then used for health policy analysis. Our results show that, in an optimistic scenario based on the status quo of deployment to intense/combat zones, estimated PTSD prevalence among veterans will be at least 10% during the next decade. The model postulates that during wars, resiliency-related policies are the most effective for decreasing PTSD. In a postwar period, current health policy interventions (e.g., screening and treatment) have marginal effects on mitigating the problem of PTSD, that is, the current screening and treatment policies must be revolutionized to have any noticeable effect. Furthermore, the simulation results show that it takes a long time, on the order of 40 years, to mitigate the psychiatric consequences of a war. Policy and financial implications of the findings are discussed.

## Introduction

Post-traumatic stress disorder (PTSD) stands out as a major mental illness, and is becoming a serious public health challenge. Currently, more than two percent of the US population (about 7.7 million people) are known to suffer from PTSD, and eight to nine percent of the US population reports experiencing lifetime PTSD [1]. In the military context, it is estimated that 11% to 20% of US military personnel who served in Iraq or Afghanistan have diagnosed or undiagnosed PTSD [2].

PTSD is the result of experiencing a traumatic event during the war such as combat, or a non-war traumatic event such as a terrorist attack, family violence, sexual assault, or serious injury [3]. Although the majority of PTSD cases in the US are caused by non-combat trauma [4, 5], the lifetime prevalence of the disorder is higher in combat-exposed cases [6]. PTSD is highly comorbid with other psychological effects or mental illnesses that can occur following trauma, including depression [7, 8], anger and violence [3], guilt and shame [9, 10], substance abuse [11, 12], and suicidality [13]. Individuals with PTSD continue to experience the psychological effects of trauma, including re-experiencing symptoms, avoidance of similar stimuli, negative cognition and mood, and increased physical arousal, long after being removed to a safe environment [14]. They may also suffer a wide range of consequences of revealing their problems, such as a higher likelihood of losing jobs or being discriminated against in the workplace, social exclusion, lower income, difficulties in renting residences, exclusion from social communities, legal difficulties [15, 16]. In addition to the patients themselves, family members, friends, community members, colleagues, and employers are also indirectly affected by PTSD.

Despite the importance of problems related to PTSD among US military personnel and veterans and recent improvements in diagnosis and treatment of the illness [17, 18], little is known about effective policies for prevention. Purtle [19] noted that ‘PTSD policy research’ is undeveloped, although knowledge about PTSD has grown over the last four decades. We believe there are three major barriers to developing effective policies:

- PTSD is a multi-organizational challenge [16]. In simple terms, patients’ family members, employers, colleagues, communities, and neighborhoods are often involved in cases of PTSD. At the macro level, larger entities such as the military, the healthcare system, the Department of Veterans Affairs (VA), and government organizations are concerned and involved with the problems of PTSD. These stakeholders have different preferences and incentives. Focusing on one organization or a specific stage of patients’ lives can result in shifting the burden to another organization rather than addressing the main roots of the problem. Most past policy studies of PTSD have focused on one sector, usually a single organization, and have not studied the consequences of the policies on other sectors.
- Similar to other dynamically complex problems, there are long delays between causes and effects—e.g., the interval between trauma exposure and diagnosis is delayed as a result of various barriers to entry into the mental health system—making current policies inadequate or ineffective.
- While there has been increased attention in the literature, there are still uncertainties about the prevalence of PTSD. Moreover, the performance of screening procedures and the accuracy of diagnosis are questionable [20]. For instance, screening procedures are usually self-reported surveys, and subjective answers can suffer from errors or be intentionally under-reported to avoid social stigma of the illness or exaggerated for secondary gain motives (e.g., disability compensation) [16, 21]. The effect of social stigma in the screening procedures is particularly important. Research shows that patients suffer a wide range of consequences of revealing their problems, such as a higher likelihood of losing jobs or being discriminated against in workplaces, lower income, difficulties in renting a residence and exclusion from social communities [15]. Consequently, the perception of possible discrimination and other consequences of being labeled as mentally ill can affect the behavior of individuals going through the screening process.

Therefore, the importance and complexity of PTSD raise critical questions: What are the trends in the population of PTSD patients among military personnel and veterans in the postwar era? What policies can help mitigate PTSD? What are the healthcare cost implications of potential policies? Furthermore, research shows that the complexity of a dynamic problem such as PTSD, which includes potential delays between causes and effects, is beyond the understanding of the human brain [22, 23]. From this complexity emerges the use of systems science and simulation-based policy analysis [24, 25], particularly system dynamics modeling [26, 27]. While systems models of different health-related problems have been developed [28–32], this study is the first to develop a system dynamics simulation model of PTSD, a model that includes both military personnel and veterans in a “system of systems”—a novel aspect of this study.

The military and VA have different purposes and functions, while the bigger system (the system of systems) is expected to have one general health-related objective which, presumably, is to improve the health of its members (i.e., military personnel and veterans). However, because the military and VA are embedded in the larger system, some of policies implemented in one of these two sub-systems may have positive ‘local’ impacts but create further problems in the larger system. Many policies implemented at the military level will potentially influence (and may have side effects on) veterans and the VA. This is mainly driven by the ‘interconnections’ between the two sub-systems as well as ‘feedback delays’; together, these increase complexity and create unexpected consequences over the long haul, such that by the time a problem becomes apparent, it might be unnecessarily difficult to solve. Without the systems-thinking lens, the behavior of the system of systems can jump without warning into a kind of mode that not only was not seen but also was not expected before (for more discussion on systems thinking, see [33]).

The systems approach in this study helps hone understanding of the parts of the system of systems and consider interconnections between them, and allows us to ask “*what-if*” questions about possible future behaviors and consequences of current policies. This analysis helps us project multi-year consequences of PTSD workloads and costs under a wide variety of what-if scenarios, ranging from those largely outside the control of the PTSD system, such as the intensity of engagements in future wars, to those under the control of the PTSD system, such as increasing resiliency to PTSD and the use of improved screening or new evidence-based treatments.

To deduce how this complex system of systems operates, we studied its components (i.e., the military and the VA) and their interconnections, and developed a model that replicates the historical behavior of the system of systems. We then use the model to conduct simulation-based what-if experiments. The rest of this article presents the data and modeling and the results of policy analysis. More detailed discussion about the data, model parameters, formulations, calibration, and validation, along with sensitivity analysis, can be found in S1 File.

## Data and Modeling

**Fig 1:**
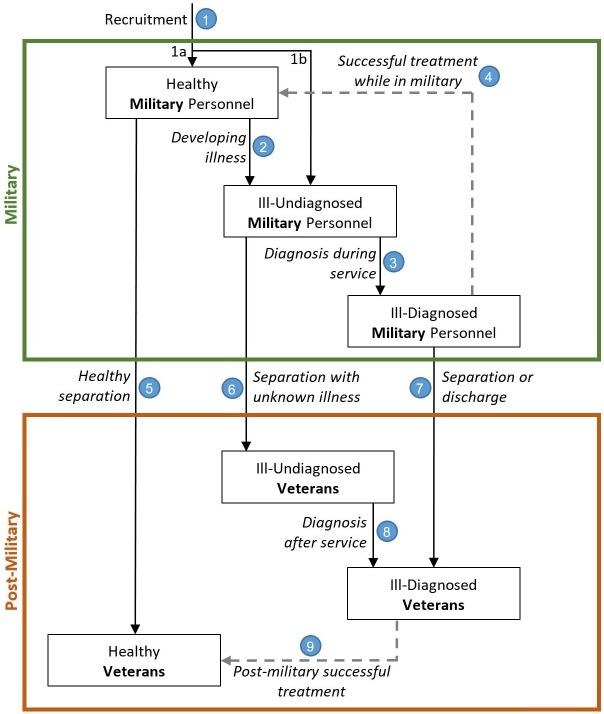
A simplified representation of the PTSD model of the “Military/Post-Military” system. All six stages have an outflow of death included in the model, but for the sake of simplicity here, they are not shown in the figure. Successful treatment rates are illustrated with dashed lines.

We used time series datasets (2000–2014) publicly available from the Department of Defense, the Institute of Medicine, the Department of Veterans Affairs, and other sources (see S1 File, Section 1) to set the base run scenario and fit the model to the data. We also extensively used the literature as a source of model parameters (see S1 File, Section 2). Our model generally presents the flow of people from recruitment into the military and from military service to the post-military stage. The basic structure of the model is illustrated in Fig 1. The model contains two major sections (large boxes): military and post-military. Each section has three stages (shown in small boxes) connected by nine potential pathways (arrows).

The two major sections of Fig 1, military and post-military, are described below.

### Military

First, people enter the military through recruitment (path 1). The model divides people in the military into three categories: healthy, ill-undiagnosed, and ill-diagnosed. The majority of the new enlisted are PTSD-free and are in the healthy military category (path 1a); however, there is a chance of recruiting people with a history of PTSD (see [34, 35] for more discussion), which is captured in path 1b (see Table B in S1 File for more information). We particularly consider path 1b in the model, because in an effort to hit recruitment targets, the military may lower criteria and recruit individuals with pre-enlistment trauma history. One might argue that the actual numbers are small, but these individuals can show up as a portion of demand for VA services related to trauma, even though their PTSD was not deployment-related.

Healthy military personnel move to ill-undiagnosed if they develop PTSD as a result of trauma (path 2). In this stage, they do not manifest any symptoms of PTSD. Military personnel move from ill-undiagnosed to ill-diagnosed when they exhibit symptoms and are diagnosed (path 3). The population of ill-diagnosed may separate or get discharged, moving to the post-military (path 7), or receive successful treatment and move back to the healthy sub-population (path 4). However, given the delays between trauma exposure and diagnosis, the PTSD problems may arise after the separation from the military (path 6).

### Post-military

Military personnel leave the military and become veterans through three paths: healthy (i.e., mentally healthy) separation (path 5), separation with unknown illness (path 6), and separation or discharge with known illness (path 7). Similar to the military section, we divide the population in the post-military into healthy, ill-undiagnosed, and ill-diagnosed. The ill-undiagnosed veterans move to the ill-diagnosed subpopulation when they are diagnosed with PTSD (path 8). Successful treatment in the post-military stage can move these ill individuals back into the healthy population (path 9).

Furthermore, in order to consider the effects of recent and long-term untreated PTSD prevalence, the population of diagnosed PTSD veterans is separated into two parts: Iraq and Afghanistan, and pre-2000 era veterans (Table A in S1 File).

The model follows the described structure. For each state variable (box variable, so-called ‘stock’ variable), the value is mathematically represented by the integration of inflow(s) minus outflow(s), following system dynamics method [22]. In each of the nine small boxes in Fig 1, a small proportion of the population die; this is included in the model, but for the sake of simplicity, it is not shown in Fig 1. Detailed model formulations as well as numbers used in the model are fully documented in S1 File (Section 3), and follow the preferred model reporting requirements (PMRR), which is a guideline for reporting simulation-based studies (see [36] for more information about PMRR).

### Model calibration and validation

The model is calibrated to the data using the partial model calibration method [37, 38], through which unknown parameters are estimated; see Section 4 in S1 File for the estimation results and more discussion. While the developed model is complex and encompasses various details about military personnel, it is still a simplification of reality; as such, it should be carefully tested and validated for the purposes of the modeling project. To build confidence in the usefulness of the model, we conducted various tests used in system dynamics modeling, such as tests of behavior validity [39], unit consistency, and equation robustness in extreme conditions [26]. Moreover, in the formulation of equations, we first tested them against different input values to ensure that the logic portrayed in the data was represented [38, 40]. Then we calibrated the model to the data. And finally, behavior reproduction tests were conducted to evaluate the ability of the model to reproduce key behaviors observed in datasets (such as the trends of PTSD patients in the military, combat-related PTSD diagnosis rate in the military, PTSD patients in VA facilities, PTSD diagnosis rate in VA facilities, pre-2000 veterans with PTSD, Iraq and Afghanistan veterans with PTSD, PTSD costs in the military, and PTSD costs in VA facilities), which helped build further confidence in the model’s usefulness [41].

Various sensitivity analyses showed that simulation results are relatively robust for considerable changes in estimated parameters, assumptions in the model, and data errors (i.e., some inconsistency among various data sources). For more information on model validation and sensitivity analysis of the estimated parameters, see S1 File, Section 5.

## Policy Analysis Results

Our policy analysis results are divided in two sections: First, we discuss the effects of future engagements in wars; second, we present the effects of improvements in diagnosis, treatment, and prevention in the military, and study their consequences not only on the military but also on the VA and the total system. To distinguish these policies in the following sections, we call the future engagements in wars “scenarios” and the improvement interventions “policies.”

### Scenarios based on future engagements in wars

We consider three scenarios for future engagements in wars:

- Scenario 1: Minimum deployment to intense/combat zones (1% of military personnel); this was the status quo in 2014 (see Figure A in S1 File for more information);
- Scenario 2: Twice the current policy, i.e., 2% deployment to intense/combat zones.
- Scenario 3: Significantly larger than the current policy, i.e., 5% deployment to intense/combat zones.

**Fig 2:**
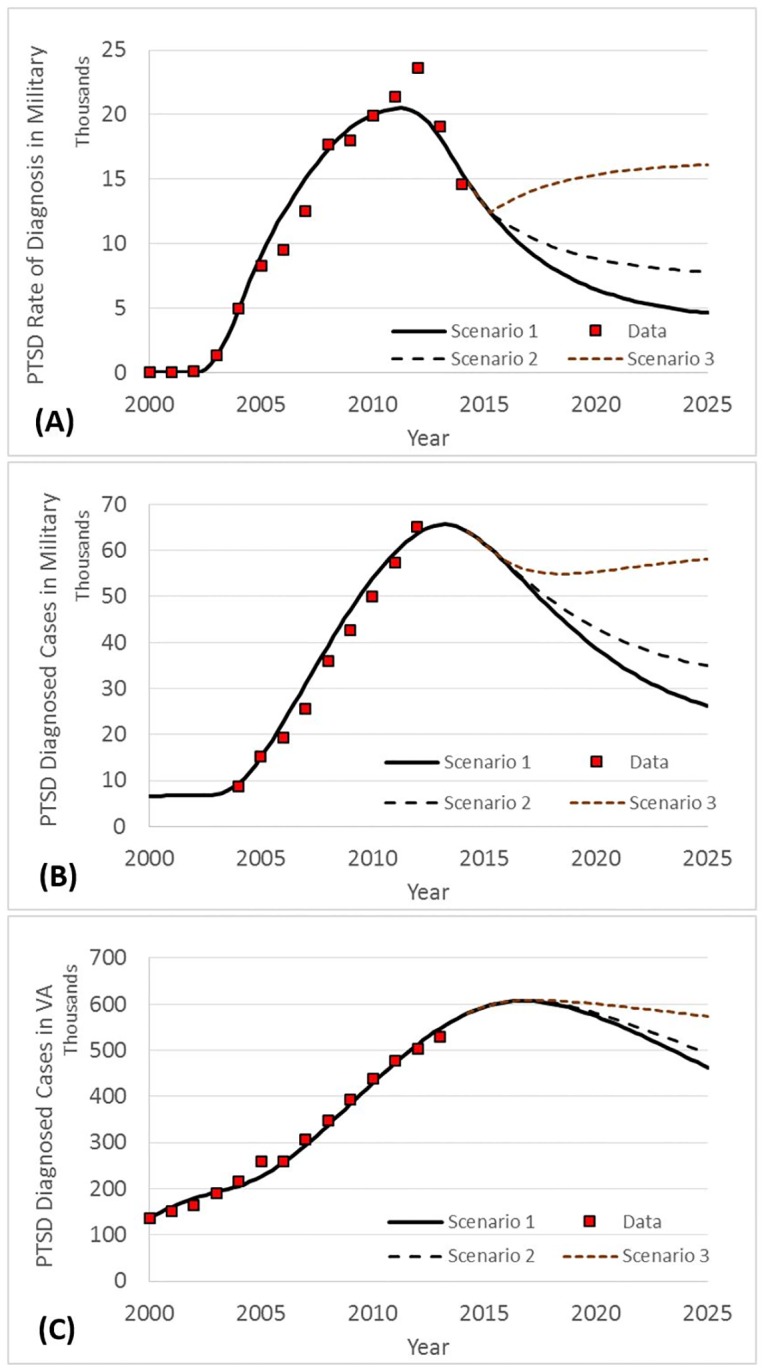
Historical data vs. simulation results, and scenarios based on future engagements in wars. (A) Diagnosis rate in military [new cases per year]. (B) Diagnosed cases in military. (C) Diagnosed cases in veterans.

As a baseline for comparison, from 2001 to 2014, on average, 6.6% of US military personnel were deployed annually to combat zones, a rate that reached a maximum of 10.8% in 2008. Fig 2 presents the base run simulations of the model for diagnosis rate in the military through the historical period, starting in 2000, and then in the future through 2025. In addition to simulation outputs, historical data (2000–2014) is also presented in Fig 2; it can be seen how closely the simulated results are a fit with the historical data.

Fig 2-A depicts the diagnosis rate in the military, which is annual new cases. As the figure shows, the number of new cases of PTSD has been declining since 2013, which is mainly due to the decreasing number of troops in Iraq and Afghanistan in recent years. The future trend, however, is very sensitive to US involvement in future wars, represented by the three scenarios. As depicted in Fig 2-B, the population of people with PTSD in the military significantly declines over time, reaching 28,000 in scenario 1 and 36,000 in scenario 2in 2025. In scenario 3, PTSD prevalence in the military increases greatly; diagnosed cases are estimated to be 58,000. Fig 2-C presents the PTSD population among veterans. Overall, the population of patients among veterans declines very slowly in comparison to the military, since people remain in the post-military stage for a long time (basically until death). Despite decreasing deployments, new cases will be diagnosed every year among veterans, because there is a delay between symptom onset and presenting at a clinic for mental health services, revealing the long-lasting effects of wars. Under scenario 3, which assumes more US troop involvement in future wars, the population of patients among veterans stays relatively constant at around 600,000 over the next decade.

We also conducted two additional analyses with the model: annual costs of PTSD and the delay in mitigating the psychological effects of a war.

#### Annual costs

**Fig 3:**
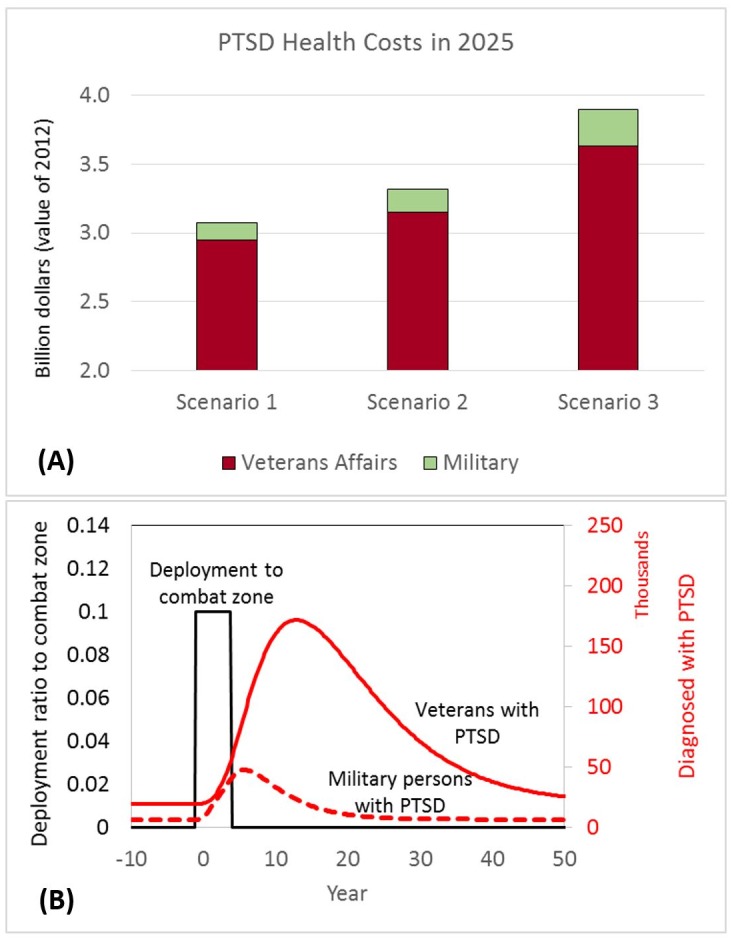
Cost projection and inertia analysis in the military and post-military systems. (A) PTSD health costs in 2025, based on the dollar value in 2012. (B) A counterfactual analysis to measure the effects of a short-term war (between years zero and five) on PTSD prevalence in the military and among veterans.

Fig 3-A presents our estimation of direct annual costs of PTSD for the military and VA healthcare systems (based on the dollar value in 2012). Cost estimation was performed based on the assumption of constant costs per PTSD patient in the military and VA over the next decade. Average cost per patient was extracted from a 2014 report by the Institute of Medicine: $4,500 and $6,244 per PTSD patient in the military and VA, respectively [20]. Accordingly, in scenario 1, estimated healthcare costs for the military and VA were $125 million and $2.95 billion, respectively. In scenario 2, these estimates rose to $164 million for the military and $3.15 billion for the VA. In scenario 3, the estimates reached $264 million for the military and $3.63 billion for the VA.

#### Delay in mitigating the psychological effects of a war

The simulation runs discussed above captured various factors that occurred during the period 2000 to 2014. Here, we ran a counter-factual simulation from a steady-state condition, presented in Fig 3-B. The purpose of this simulation was to analyze the inertia in the system and measure how long it takes to mitigate the effects of a hypothetical 5-year war with 10% troop deployment (around the maximum deployment in Iraq). We used the estimated parameters from the model, but isolated all exogenous variables (i.e., variables assumed to change from outside our model). The results, presented in Fig 3-B, show the long delay in mitigating the psychological effects of a war. Controlling for treatment, screening, and training policies, Fig 3-B shows that it takes about 40 to 45 years for the veteran population to become PTSD-free. This represents the long-lasting effects of a war. Furthermore, Fig 3-B shows that the peak for the PTSD population among veterans emerges about six years after the war ends, and the PTSD population is much higher among veterans than in the military.

### Interventions based on diagnosis, treatment, and prevention

Similar to most other health interventions, our policies focus on improving diagnosis, treatment effectiveness, and prevention. We also had a base run scenario—without any interventions—considered as control group. The overall goal in this section is to study how policies implemented in the military will affect not only military personnel but also veterans and the total system.

In Policy 1, improving diagnosis, the focus was on screening. We formulated this policy by doubling the screening sensitivity in the military (i.e., the annual rate at which undiagnosed PTSD is diagnosed). Given that there is no data on how many military personnel have PTSD and are undiagnosed, we estimated the rate at which undiagnosed military personnel are diagnosed through model calibration. In a nutshell, the rate of diagnosis during service (people/year) is a product of the fractional rate of revealing symptoms (a constant coefficient, estimated to be 0.043/year) and the population of ill-undiagnosed military members (a dynamic variable, unit: person); see S1 File for more information about the formulation, estimation, and sensitivity analysis on the estimation. In this policy, the fractional rate of revealing symptoms is doubled, representing higher speed of diagnosis, and the effect is analyzed.

In Policy 2, the focus was on improving treatment, and we tested the effects of doubling the successful treatment rate while PTSD patients are in the military. Despite the increased attention in the literature, lack of comprehensive outcome data on treating combat-related PTSD [42] and high heterogeneity among different findings [17] are still significant challenges. We therefore estimated the successful treatment rate through model calibration. The rate of successful treatment (people/year) is a product of fractional treatment rate (a constant coefficient, estimated to be 0.125/year) and ill-diagnosed military members (a dynamic variable, unit: person); see S1 File for more information. In this policy test, we doubled the fractional treatment rate, and the effect was examined.

In Policy 3, we tested the effects of improvement in PTSD prevention. This policy represented effective training programs that might improve the resilience of military personnel to PTSD (i.e., the ability of individuals to maintain a stable equilibrium, which can be achieved through psychological pathways such as hardiness, self-enhancement, repressive coping, and positive emotion—see [43] for more discussion on each pathway). It should also be noted that non-psychological factors, such as genetic and neurobiological dysfunctions; are also dimensions which affect resilience [44]. Overall, we formulated Policy 3 by decreasing the average likelihood of getting PTSD after trauma. We formulated a condition where resiliency to trauma was doubled, meaning that the chance of developing PTSD after experiencing trauma was halved (the base likelihood of 17% [45] is considered in our model).

**Fig 4:**
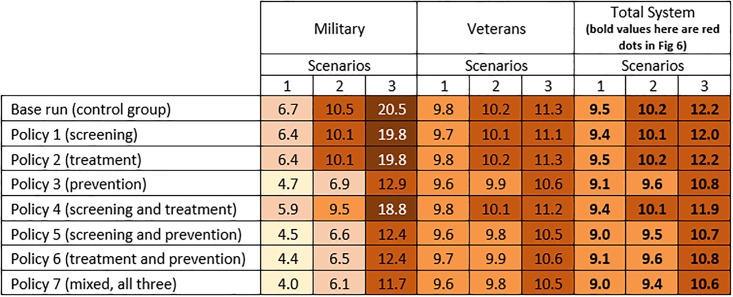
Simulation results for PTSD prevalence in 2025 for the military, the VA, and the total military-VA system under the three scenarios and interventions (Policy 1–7). Note: See the instructions under Fig 5 on how to read the figure.

In Policies 1–3, a single model input was changed and model outputs were analyzed accordingly; however, we tested all combinations of these three policies, which created four additional policies (i.e., Policies 4–7, see Fig 4). It should be noted that our model is fully reported (see Supporting Information), and if future researchers find different base line values (e.g., a different base line value for the average likelihood of developing PTSD after trauma), or prefer to test different changes in the base line (e.g., instead of doubling the resilience rate to trauma, increase it by 50% or so), they can simply implement the changes in the model, run the simulation, and observe the results. In order to do this, the model can be run offline using the Vensim software program (by Ventana Systems, Inc.) or online without any software requirements at http://jalali.mit.edu/ptsd-simulation—the offline version includes more features (see S2 File); the online version is developed in a more interactive environment.

**Fig 5:**
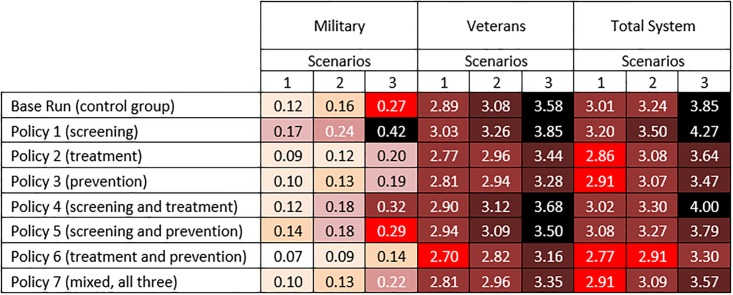
Simulation results for PTSD healthcare costs in 2025 for the military, the VA, and the total military-VA system under the three scenarios and interventions (Policy 1–7). For each sector (military, veterans, and total system), 24 combinations of the three scenarios and seven policy interventions along with the base run are presented.—Darker colors represent larger numbers.—One way to read the figures is to compare the effects of policies in a scenario (e.g., scenario 1). In Fig 4, e.g., in the military under scenario 1 (little involvement in future wars), PTSD prevalence was estimated to be 6.7% in the base run. If policies 1 or 2 were implemented, PTSD prevalence decreased slightly to 6.4%. However, with Policy 3, the prevalence decreased to 4.7%. In Figure 5, e.g., in military, under scenario 1, PTSD healthcare costs are estimated to be $0.12B in the base run. Going down in the same column, e.g., for policies 1 through 3, the PTSD healthcare costs in the military are estimated to be $0.17B, $0.09B, and $0.10B, respectively.—Another way to read the figures is to compare different scenarios over a policy.—Based on Fig 4, policy 7 results in lower prevalence and based on Figure 5, policy 6 results in the most cost saving.

**Fig 6:**
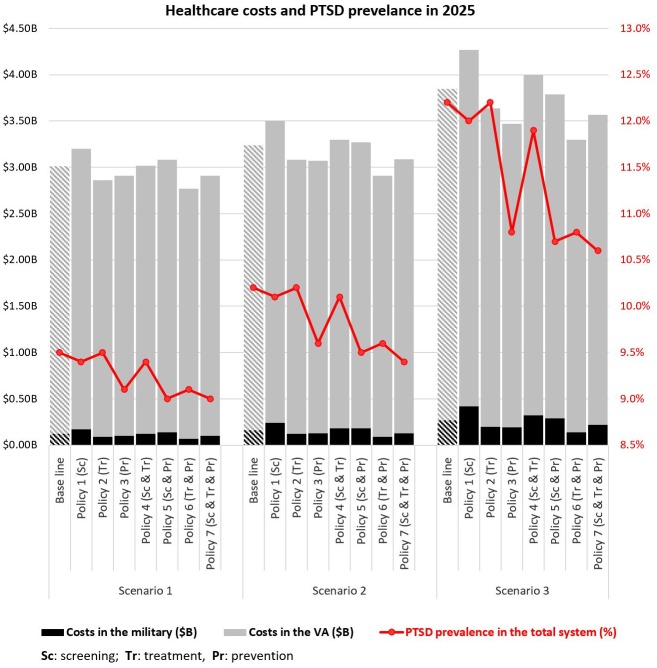
Healthcare costs in the military and the VA, and PTSD prevalence in the total system in 2025.

For each sector (military, veterans, and total system), we analyzed Policies 1–7 along with the base line vs. the three scenarios of future involvement in wars; therefore, 24 combination model outputs are presented. We used two major outputs as policy measures: PTSD prevalence and PTSD healthcare costs. Figs 4 and 5 presents these policy measures for 2025. Figs 4 and 5 present PTSD prevalence (%) and healthcare costs (in billions), respectively. Values in both figures are color coded to emphasize the magnitude of the numbers and provide comparisons across all conditions (darker colors represent larger numbers). Fig 6 also provides a more visual presentation of the results in Figs 4 and 5.

#### PTSD prevalence

Based on Figs 4 and 6, it is notable that Policy 1 (sole focus on screening) and Policy 2 (sole focus on treatment) had minimal effect compared to the corresponding values in the base run. The main reason for achieving this result for Policy 1 is that with the current effectiveness of treatments (argued to be low; see [17] for more discussion), a sole focus on screening may only lead to finding more PTSD-positive individuals who are not treated successfully. In Policy 2, a focus on treatment alone also does not help much either, because many PTSD patients are not diagnosed or are late-diagnosed. Consequently, the combination of Policies 1 and 2 (i.e., Policy 4) slightly decreased the prevalence rate compared to the base run. However, the prevalence rates of Policy 3 (sole focus on prevention) and any other combination of policies which include the focus on prevention (i.e., policies 5–7) were considerably lower—e.g., under Scenario 1 in Fig 6, compare the red dots of policies 3 and 5–7 to those of the base line and policies 1–2 and 4. The same pattern—where policies with a focus on prevention decreased the prevalence noticeably—is also achieved for the military, the VA, and the total system across all three scenarios (see Fig 6).

Based on the results in Fig 4, it is important to note that under scenario 1, the effects of all policies were still limited to the small population of military personnel with PTSD, and the VA will be still facing a large number of PTSD patients (between 9.6% and 9.8% across all policies).

#### PTSD healthcare costs

Policy 1 (sole focus on screening) not only costs the military and VA more than the base run, but it is also more expensive than all other policies across the three scenarios (see Fig 6). As discussed in the previous section, under Policy 1, since more people are screened, more PTSD positives are diagnosed. However, without advancements in treatment, screening improvement only increases the demand for care. In other words, because the successful treatment rate is already small, this policy mainly results in higher demand for treatment and a higher rate of discharge from the military. Consequently, not only do costs for the military increase, but also, unexpectedly, costs (and demand for care) for the VA rise due to more military discharges, an example of shifting the burden in a complex system of systems. Thus, Policy 1 alone can make us worse off in cost-related measures and also has minimal effect on PTSD prevalence (see Figs 4 and 6).

Policy 2 (sole focus on treatment) decreases the costs in comparison to the baseline. This policy mainly helps treat already diagnosed patients at a more successful pace; however, it also has a limited effect on PTSD prevalence, as many patients remained undiagnosed. Overall, Policy 3, which focuses on prevention by increasing the resilience to PTSD, results in costs similar to Policy 2; however, with more involvement in future wars, Policy 2 becomes more costly—compare the costs of policies 2 and 3 across the three scenarios in Fig 6 (a similar pattern can also be seen for policies 4 and 5).

Among the policies with a focus on a combination of screening, treatment, and prevention (i.e., policies 4–6), Policy 6 (focus on treatment and prevention) has the lowest costs. In fact, Policy 6 has the lowest costs in comparison to all other policies as well as the base line; however, its PTSD prevalence rate is relatively higher than that of policies 5 and 7 (see Fig 6). Policy 7, which is a combination of screening, treatment, and prevention, is similar to policies 2 and 3 in terms of costs, but it has the lowest PTSD prevalence rate compared to all other policies, which makes it a potential choice for mitigating PTSD. One might argue that Policy 6 is cheaper and its PTSD prevalence rate is not much different from the prevalence rate of Policy 7. This might seem reasonable, but in fact, it is missing improvements in screening that are critical to intervention with diagnosed PTSD patients, and without which many individuals may remain undiagnosed in the system.

## Discussion and Conclusions

We developed a systems model of the population of military personnel and veterans affected by post-traumatic stress disorder (PTSD) and compared the results with the historical data for 2000–2014. Then, the model was used to forecast the trends for the next decade under several scenarios of US involvement in future wars. The major insights from the model are: 1) The population of patients and system costs are very sensitive to US involvement in future wars, and screening and treatment policy interventions have marginal effects in comparison; 2) In an optimistic scenario based on the status quo (about 1% deployment of military personnel to intense/combat zones in 2014) and assuming that no war will happen in the next decade, estimated PTSD prevalence among veterans in 2025 will be 10%; 3) During wars, resiliency-related policies are the most effective for decreasing PTSD; in a postwar period, there is no silver bullet for overcoming the problem of PTSD, and the current screening and treatment policies must be revolutionized to have any noticeable effect; and 4) It takes a long time, on the order of 40 years, to mitigate the psychiatric consequences of a war.

It should be noted that our study objective was to conduct ‘what-if’ analyses. In terms of treatment, we analyzed the long-term effects if the current effective treatment rates are doubled—we did not intend to study ‘how to’ improve treatment. There is already increasing attention in the literature to discussing these how-to concerns, e.g., see [43, 44, 46–48] for increasing resiliency to PTSD (prevention), [49–52] for improving screening, and [53–57] for enhancing treatment. There still remains a lot to do on this how-to stream of research, yet little attention has been paid to the long-term effects of the policies we studied, particularly in a system of systems that includes both the military and VA.

Our findings may help the military, the VA, and other government entities identify more effective strategies and also interact more effectively with one another. PTSD is a multi-organizational problem, and focusing on one organization or a specific stage of patients’ lives can result in shifting the burden to another organization. We would also like to clarify that all cost estimations in the study are based on average expenditure per patient in military and VA healthcare facilities. The actual costs for each patient are much higher, including the cascading effects of the illness on personal life [16].

### Limitations and future studies

This study is subject to some limitations. The major limitation concerns exogenous variables, i.e., variables assumed to change from outside our model. We assumed deployment to war is an exogenous variable for a model of a healthcare system. In simple words, changes in the number of PTSD patients at the levels we are discussing (10% to 20%) are not likely to influence politicians’ decisions about entering wars. In an ideal world, we hope that with understanding of high PTSD prevalence and more social pressure, involvements in future wars will decrease and the military will be downsized. With a smaller military, one can be even more selective and recruit more resilient military personnel.

We also assumed that PTSD costs are not feeding back to decisions about involvement in future wars. We think that since the direct costs of PTSD (inpatient and outpatient costs) are small in comparison to all military costs, our assumption is fairly reasonable. Moreover, we did not include a variety of factors at the macro level, such as the US economy or politics of elections. These variables are more important for modeling involvement in wars. The purpose of our model was not analyzing the dynamics of wars, but a specific consequence of wars under various scenarios.

Moreover, our work mainly describes the model as a flow process with few but relevant feedback loops. Future modeling studies could incorporate more feedback loop mechanisms. More dynamic factors can also be considered. Future research could examine how barriers to care such as comorbidity (i.e., the presence of comorbid mental and physical health conditions) and social stigma (i.e., labeling and social exclusion associated with mental illnesses) interfere with both diagnosis and treatment of PTSD and therefore hamper the outcomes of the policies—see [18] for more discussion. Another topic could be how improvements in treatment and screening over time could eventually result in different behavior of the model outputs.

One should also take into account that the proportion of female service members has increased and may continue to do so, and females may have higher likelihood of sustaining PTSD in a combat situation. Furthermore, we followed the usual dichotomous status for treatment of PTSD, but we acknowledge that mental illnesses almost always exist on a continuum, and imposing an on-off switch is relatively arbitrary. In addition to addressing our study limitations, future studies could also further validate our findings.

Despite these limitations, we hope the current study provides a first systematic step towards better understanding the consequences of PTSD policies. Our model is fully documented, not only for investigating various policies and analyzing the results over the long haul, but also for further development and replications. The model is available to be run online at http://jalali.mit.edu/ptsd-simulation. The historical data and model assumptions (embedded in model parameters and equations) are continually refined at this web address.

## Supporting Information

S1 File Supporting Information Document. This document includes five sections: 1) Data–Time Series, 2) Data–Parameters, 3) Model Formulation, 4) Model Calibration, and 5) Model Validation and Sensitivity Analysis. The document also includes supporting figures (Figures A-N) and tables (Tables A-E).
(PDF)

S2 File Supporting Information Files. This zipped file includes the simulation model (*PTSD_Simulation*.*mdl*), data file (*Data*.*vdf*), as well as a ReadMe document (*ReadMe*.*docx*). See the ReadMe document for instructions on how to use the model and data. The simulation can also be run online without any software requirements at http://jalali.mit.edu/ptsd-simulation—*PTSD_Simulation*.*mdl* file includes more features; the online version is developed in a more interactive environment.
(ZIP)
