# Supplementary material for: A Dynamic Model of Post-Traumatic Stress Disorder for Military Personnel and Veterans
Source: PLoS One. 2016 Oct 7;11(10):e0161405. doi: 10.1371/journal.pone.0161405 (PMC5055362; doi:10.1371/journal.pone.0161405)
Supplement: S1 File — This document includes five sections: 1) Data–Time Series, 2) Data–Parameters, 3) Model Formulation, 4) Model Calibration, and 5) Model Validation and Sensitivity Analysis. The document also includes supporting figures (Figures A-N) and tables (Tables A-E). (PDF) [file pone.0161405.s001.pdf]

Supporting Information Document for

A Dynamic Model of Post-Traumatic Stress Disorder for Military Personnel and Veterans

Navid Ghaffarzadegan[1], Alireza Ebrahimvandi[1], Mohammad S. Jalali[2*]

[1] Industrial and Systems Engineering, Virginia Tech, Blacksburg, Virginia, United States of America
[2] Sloan School of Management, Massachusetts Institute of Technology, Cambridge, Massachusetts, United States of America

[*] Corresponding author
Email: Jalali@mit.edu

Contents:

Section 1: Data - Time Series

Section 2: Data - Parameters

Section 3: Model Formulation

Section 4: Model Calibration

Section 5: Model Validation and Sensitivity Analysis

Section 1: Data - Time Series

Table A provides a summary of time series data used in this study. We present more details about these data.

Table A. Summary of time series used in the model

No	Variable	Values	Sources
**Military and Veterans Population**			
1	Military population	Figure A	Institute of Medicine (1) & D.o.D. (2)
2	Troops in Iraq combat zone	Figure A	Liu, Xiao (3)
3	Troops in Afghanistan combat zone	Figure A	Liu, Xiao (3)
4	Military recruitment	Figure B	Cronbach (4)
5	Military separation rate	Figure B	Authors' estimation using [D.o.D. (2) & D.o.D. (5)] and Cronbach (4).
6	Veterans population	Figure C	Institute of Medicine (1) & Richardson and Waldrop (6) and Bagalman (7)
**PTSD related variables**			
7	PTSD diagnosed in military	Figure D	Institute of Medicine (1)
8	PTSD diagnosis rate in military		Institute of Medicine (1) and Fischer (8)
	8.1. Diagnosis rate of deployed	Figure D	Fischer (8)
	8.2. Diagnosis rate of not-deployed	Figure D	Fischer (8)
9	PTSD diagnosed veterans (all)	Figure E	Institute of Medicine (1), Rosenheck and Fontana (9) and Hermes, Rosenheck (10)
	9.1. Diagnosed; Iraq and Afghanistan veterans	Figure E	Rosenheck and Fontana (9) and Hermes, Rosenheck (10)
	9.2. Diagnosed; pre-2000 era veterans	Figure E	Rosenheck and Fontana (9)
10	PTSD diagnosis rate of veterans	Figure E	Institute of Medicine (1)
**Cost related variables**			
11	PTSD costs in military	Figure F	Institute of Medicine (1)
12	PTSD costs in Veterans Affairs	Figure F	Institute of Medicine (1)

Military and Veterans Population

1. Military population
   Unit: Persons
   Description: The military includes active and reserve members.
   Values: Figure A
   Source: Institute of Medicine (1) data for 2004-2012. The rest of the data are extracted from D.o.D. (2).

2. Troops in Iraq combat zone
   Unit: Persons
   Description: The military personnel deployed to Iraq
   Values: Figure A
   Source: The Department of Defense report, Liu, Xiao (3)

3. Troops in Afghanistan combat zone
   Unit: Persons
   Description: Military personnel deployed to Afghanistan
   Values: Figure A
   Source: The Department of Defense report, Liu, Xiao (3)

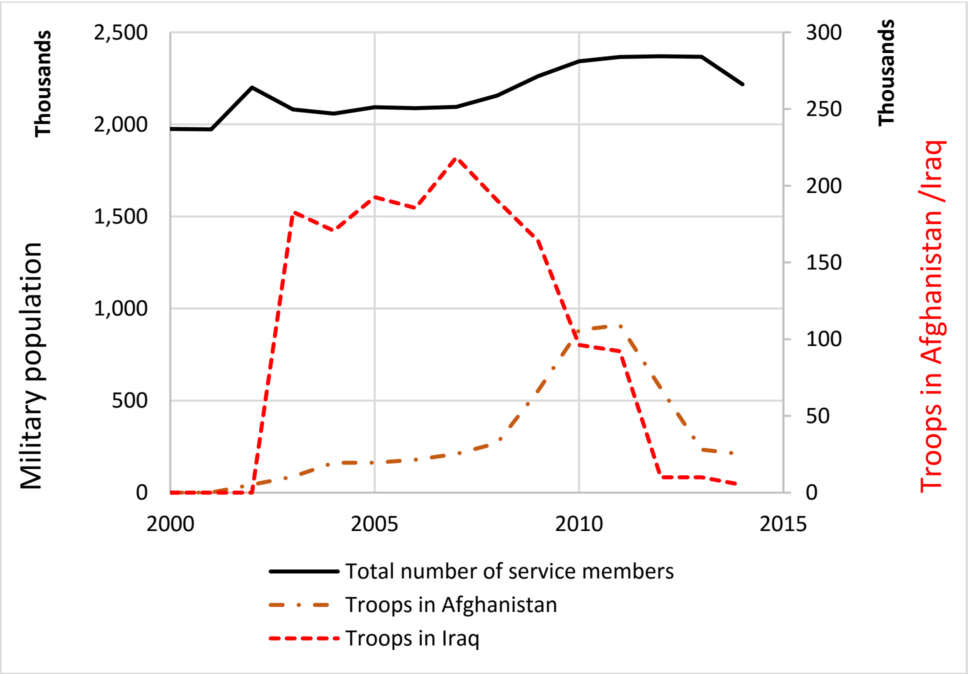

Figure A. Total number of service members in the military (left axis) and deployment to Iraq and Afghanistan (right axis) for 2000-2014.

4. Military recruitment

   Unit: Persons/year

   Description: Annual recruitment for active and reserve personnel

   Values: Figure B

   Source: Extracted from annual Department of Defense announcement for recruiting and retention for fiscal years of 2000 to 2014 D.o.D. (11).

5. Military separation rate

   Unit: persons/year

   Description: Annual exit rate (separation) from the military

   Values: Figure B

   Source: Authors' estimation from the military population and recruitment data [D.o.D. (2) and D.o.D. (5)] and Cronbach (4). For year t, we assume: Military separation rate(t) = Recruitment(t) – [Military population(t+1)- Military population(t)].

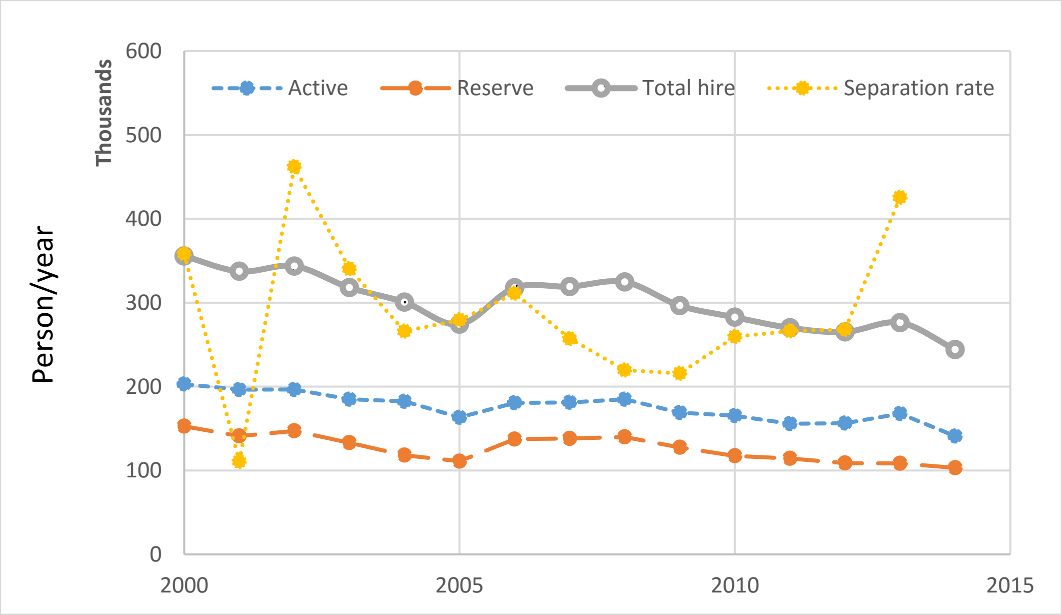

Figure B. Military hiring rate (active, reserve, and total) and separation rate for 2000-2014

6. Veterans population

   Unit: Persons

   Description: All living veterans (18 years old and older)

   Values: Figure C

Source: Institute of Medicine (1) for 2010-2012. The rest of the data are extracted from Bagalman (7) and Richardson and Waldrop (6).

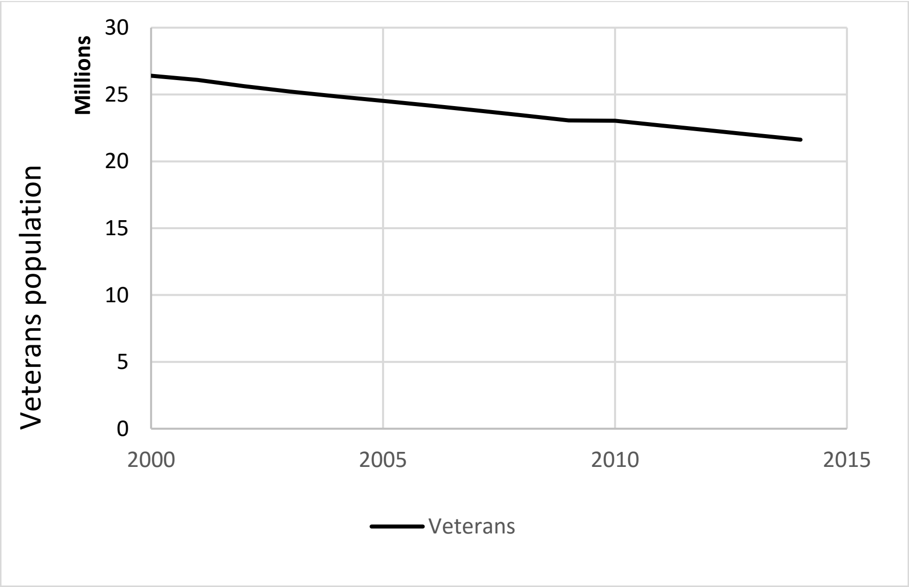

Figure C. Total number of living veterans

PTSD related variables

7. PTSD diagnosed in military

Unit: Persons

Description: Number of the military service members who are diagnosed with PTSD. The Institute of Medicine considers a military person to have PTSD if during the year he/she had one inpatient stay or two outpatient visits, at least one day apart with the diagnosis.

Values: Figure D

Source: Institute of Medicine (1) reports diagnosed military for 2004-2012.

8. PTSD diagnosis rate in military

Unit: Persons/Year

Description: Annual new diagnosis of PTSD in the military

Values: Figure D

Source: Institute of Medicine (1) reports PTSD diagnosed in the military for 2004-2012. The rest of the data are based on a congressional report by Fischer (8) with minor adjustment to make consistent with the IOM report.

Sub-categories:

8.1. Diagnosis rate among the military members deployed to Iraq and Afghanistan, extracted from Fischer (8).

8.2. Diagnosis rate among the military members not deployed, extracted from Fischer (8).

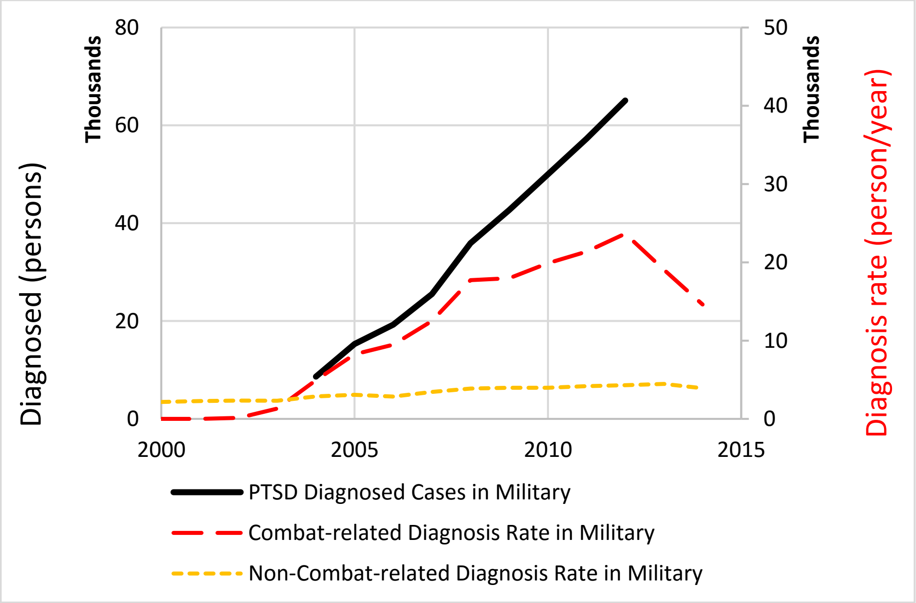

Figure D. PTSD Diagnosed in the military and Diagnosis Rate (new cases)

9. PTSD diagnosed veterans

Unit: Persons

Description: Numbers of veterans receiving veteran affairs (VA) specialty mental health services for PTSD. The Institute of Medicine considers a veteran to have PTSD if during the year before he/she had one inpatient stay or two outpatient visits, at least one day apart with the diagnosis.

Values: Figure E

Source: Institute of Medicine (1) reports data for years 2008 and 2013. The rest of the data are based on Rosenheck and Fontana (9) and Hermes, Rosenheck (10).

9.1. Veterans of Iraq and Afghanistan with PTSD

Source: Rosenheck and Fontana (9) and Hermes, Rosenheck (10)

9.2. Veterans of Pre-2000 era with PTSD

Note: We added up data on PTSD from wars prior to Afghanistan and from peace periods. A major portion of these data relates to Vietnam veterans.

Source: Data extracted from Exhibit 1 of Rosenheck and Fontana (9).

10. PTSD diagnosis rate of veterans

Unit: Persons/Year

Description: Annual new diagnosis of PTSD in VA related facilities

Values: Figure E

Source: Institute of Medicine (1) reports data for years 2008 and 2013.

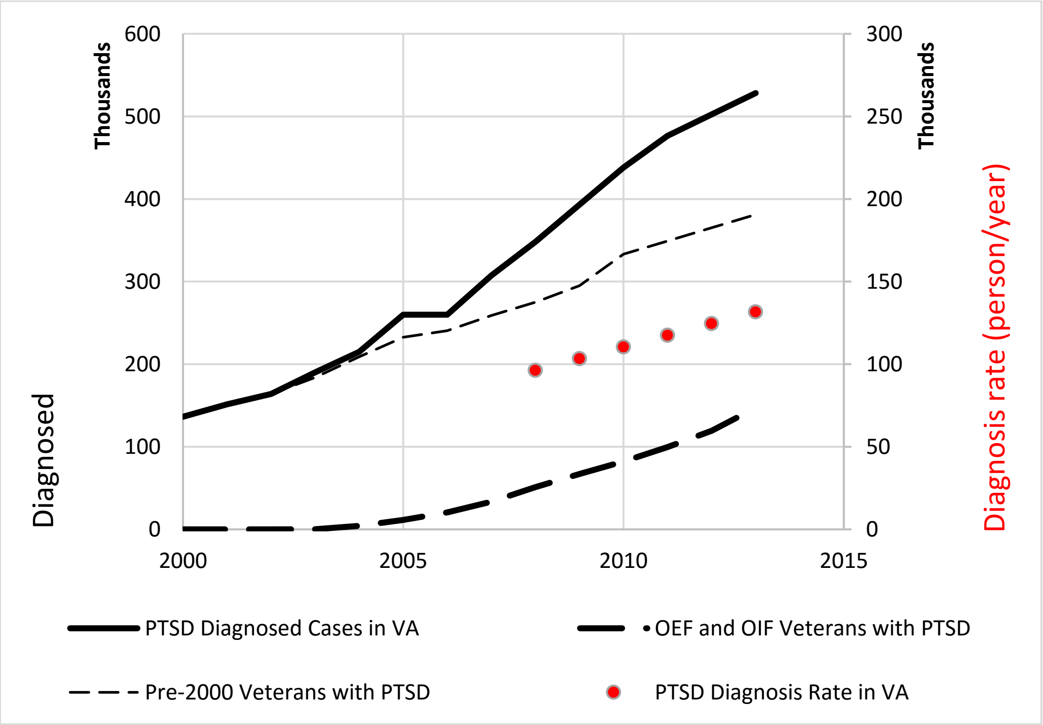

Figure E. PTSD diagnosed in VA and diagnosis rate (new cases per year)

__**Cost related variables**__

11. PTSD costs in military

Unit: $

Description: This is only direct cost associated with a person under treatment in the military. The Institute of Medicine estimates this cost based on average costs per day for

inpatient and outpatient visits related to PTSD and costs of buying drugs. The numbers are in real terms (inflation-adjusted) for the 2010 dollar value.

Values: Figure F

Source: Institute of Medicine (1)

12. PTSD costs in Veterans Affairs

Unit: $

Description: This is only direct cost associated with a person under treatment in VA. The Institute of Medicine estimates this cost based on average costs per day for inpatient and outpatient visits related to PTSD and costs of buying drugs. The numbers are in real terms (inflation-adjusted) for the 2010 dollar value.

Values: Figure F

Source: Institute of Medicine (1).

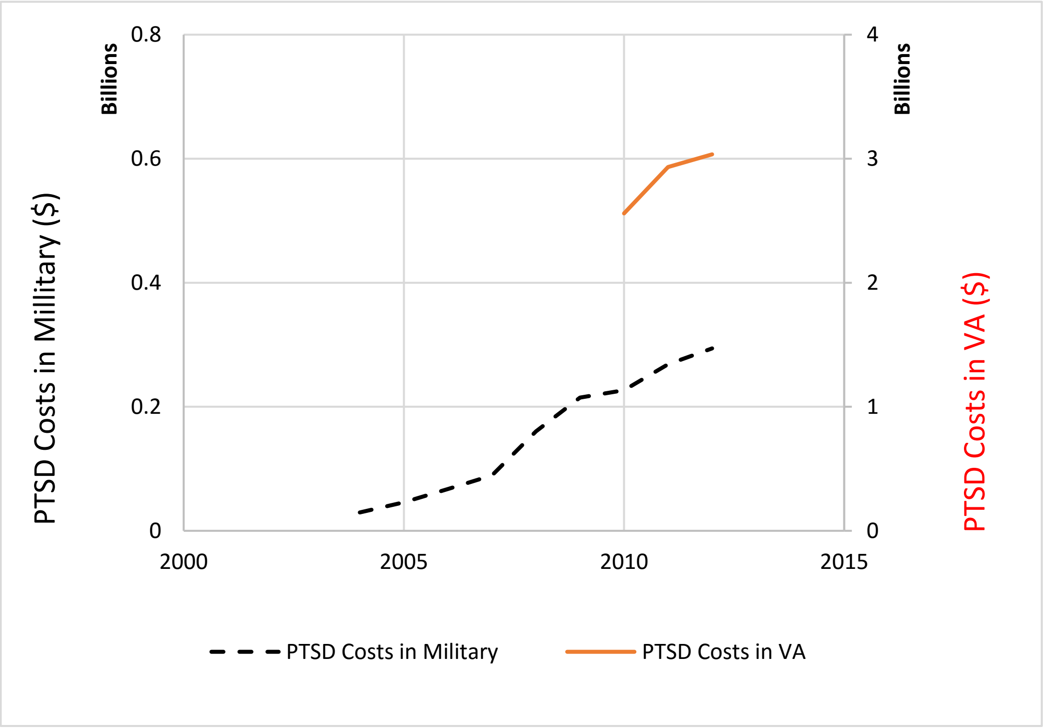

Figure F. PTSD direct costs in the military and VA

Section 2: Data - Parameters

Parameters are assumed to be constant during the entire simulation run (2000-2025). Table B provides a summary of the parameters, their values, and sources. In following, we provide more details about these parameters.

Table B. Summary of data used for model parameters

No	Parameters	Value	Sources
1	Annual separation ratio	12.5%	Based on three estimates: DeFraites and Vythilingam (12), Segal and Segal (13), and our estimation using Little's Law [14].
	1.1. Separation ratio of ill-undiagnosed	18%	Estimated using item 1 of this table.
	1.2. Separation ratio of previously diagnosed	33%	DeFraites and Vythilingam (12)
	1.3. Separation ratio of ill-diagnosed	20%	Dunbar (15)
2	Probability of getting PTSD given a trauma	17%	Martin (16)
3	Average number of traumas a deployed person experiences per year (traumas/year/person)	4.5	Weighted average of items 3.1 and 3.2 from Hoge, Castro (17)
	3.1. traumas/year/person in Iraq	6.21	Hoge, Castro (17)
	3.1. traumas/year/person in Afghanistan	3.02	Hoge, Castro (17)
4	Unhealthy recruitment ratio	0.01	Monahan, Hu (18)
5	Normal fractional rate of developing PTSD for non-combat related reasons	0.1%	Kilpatrick, Resnick (19) and Organization for Economic Cooperation Development Staff (OECD) (20).
6	Average cost per PTSD patient in military after 2012	$4,500	Institute of Medicine (1)
7	Average cost per PTSD patient in VA after 2012	$6,244	Institute of Medicine (1)
8	Fractional death rate for veterans (1/year)	0.023	Authors' estimation based on USA Social Security Administration (21)
	8.1. Fractional death rate for Iraq and Afghanistan veterans	0.023	Authors' estimation based on USA Social Security Administration (21)
	8.2. Fractional death rate for veterans non-deployed	0.023	Authors' estimation based on USA Social Security Administration (21)
	8.3. Fractional death rate for pre-2000 veterans	0.030	Authors' estimation based on USA Social Security Administration (21) and Richardson and Waldrop (6)

No	Parameters	Value	Sources
9	Fractional death rate for military personnel	0.010	Authors' estimation based on USA Social Security Administration [22] and [23]

1. Annual separation ratios

Unit: 1/year

Description: This is the annual exit ratio from the military for different subpopulations, described in sections 1.1-1.3 below.

Value: 12.5%

Source: DeFraites and Vythilingam (12) estimated annual separation rate of 15% for the military personnel population. This means every year about 15% of military personnel leave the military. If we take this estimation, the average duration of service should be slightly less than 7 years. This is not far from the argument of Segal and Segal (13). They state that the average length of service in the U.S. military is less than 10 years. We also used little’s law [14] to estimate the exit rates assuming relatively constant population in the military and a stable hiring rate (W=Average waiting time = Population/recruitment). This gives us W=7.8 years. Based on these sources, we assumed 8 years to be a reasonable estimate for the duration of service—for further validation, we conduct sensitivity analysis to build confidence in this assumption. This means the whole population of military personnel separates in the rate of 1/8=12.5%.

We used different methods to estimate separation ratios for different sub-categories:

1.1. Separation ratio of ill-undiagnosed: This is the population that has PTSD but not diagnosed. We assumed that the military personnel get deployed for about a year (or a year and a half) and they have some training before the deployment [1]. So it would be reasonable to assume that on average for people who have just developed PTSD in a combat zone it takes about 5.5 years (8-2.5 years) to separate. Thus separation ratio for this sub-population would be roughly 18%.

1.2. Separation ratio of previously diagnosed: Based on DeFraites and Vythilingam (12), 33% of the military personnel diagnosed with PTSD separate from the military in a year. However, a considerable portion of them (not necessarily all) are still ill and need care.

1.3. Separation ratio of ill-diagnosed: Following the quote by TSG Horoho: "The good news is 80% of those diagnosed with PTSD return to duty verifying what we in medicine have always known, PTSD is a treatable condition,” [15]. Accordingly, we believe it is reasonable to assume that 20% get discharged very soon as ill, and the rest of them (13%) get discharged in other forms, either as healthy, or ill-undiagnosed, in case they only discontinued their treatment.

2. Probability of getting PTSD given a trauma

   Unit: person/traumatic event

   Description: The average chance of developing PTSD if someone faces a trauma (with no special resiliency-related intervention)

   Value: 17%

   Source: Reported by Martin (16)

3. Average number of traumas a deployed person experiences per year

   Unit: trauma/person/year

   Description: This value varies for different wars. Hoge, Castro (17) conducted a survey on selected infantry units of deployed military members in Iraq and Afghanistan to investigate the relation of exposure to combat and mental problems including PTSD. Their survey presents that traumatic exposure rate is 4.26 among 1,962 members in Afghanistan, and 8.74 among 1,709 members in Iraq. The weighted average (weighted based on the number of members) for traumatic events among all troops in Afghanistan and Iraq is 6.35. Length of deployment of multiple deploys, by branch of service and component as of 2010 is reported to be 16.9 months [24]. Therefore, the average rate of trauma per person per year should be around 4.5 (6.34*12/16.9).

   Value: 4.5

   Source: [17] and authors' estimations

   3.1. Traumas/year/person in Iraq:

   Hoge, Castro (17) estimated 8.74 traumas per person happens during the deployment in Iraq. Based on this, annual rate will be 6.21 per person.

   3.2. Traumas/year/person in Afghanistan

   Hoge, Castro (17) estimated 4.26 traumas per person happens during the deployment in Iraq. Based on this, annual rate will be 3.02 per person.

4. Unhealthy recruitment ratio

   Unit: dimensionless

   Description: This parameter represents the ratio of new hires who are assumed to be PTSD free (they may have other illnesses).

   Value: 0.01

Source: Monahan, Hu (18) reports that the incidence of PTSD in recruit trainees among all of the service branches in 2000-2012 was 3.3 per 1,000 person-years. This will be the known cases. We assume that including unknown cases, 1% might be a reasonable estimate. Later in sensitivity analysis we change this parameter in a range of [0,0.05].

5. Normal fractional rate of developing PTSD for non-combat related reasons

   Unit: 1/ year

   Description: This is the chance that one develops PTSD while in the military for non-combat related reasons. We estimate this number from civilian lives, assuming that the average military personnel is as resilient as an average civilian and faces the same rate of traumatic conditions as a civilian experiences.

   Lifetime prevalence of PTSD among U.S. adults is 7.8 percent [19]. Life expectancy is about 80 years [20]. We roughly estimate the chance of developing PTSD about 0.078 over 80 years, equal to 0.1% per year per person. Overall this number is very low in comparison to combat related traumas which makes the model not sensitive to our estimation.

   Value: 0.1% /year

   Source: Authors' estimation using [19] and [20]

6. Average cost per PTSD patient in military after 2012

   Unit: $/person

   Description: We conservatively take the 2012 value, assuming no further healthcare cost inflation.

   Value: $4,520 per diagnosed patient. This value is a weighted average of inpatient costs and outpatient costs.

   Source: Institute of Medicine (1)

7. Average cost per PTSD patient in VA after 2012

   Unit: $/person

   Description: We conservatively take the 2012 value, assuming no further healthcare cost inflation.

   Value: $6,244per diagnosed patient. This value is a weighted average of inpatient costs and outpatient costs.

   Source: Institute of Medicine (1)

8. Fractional death rate for veterans (death ratio per year for veterans)

   Unit: 1/year

Description: We estimate this ratio based on the life expectancy of US citizens. Life expectancy of a 40 years old in the US is 40.4 years (death ratio: 1/40=0.025), and life expectancy of a 30 years old is 49.8 years (death ratio 1/50=0.02). We roughly estimate death ratio = 0.023 which also gives us a very good fit to data.

Value: 0.023

Source: Authors' estimation from data about life expectancy in the US using USA Social Security Administration (21).

8.1. Death ratio for Iraq and Afghanistan veterans: 0.023

8.2. Death ratio for veterans not deployed: 0.023

8.3. Death ratio for pre-2000 veterans: Median age of pre-2000 veterans was 57.4 in 2000 [6]. Life expectancy of a 60 years old is 22.8 [21]. Therefore, we estimated death ratio to be 0.04 (1/25).

9. Fractional death rate for military personnel (death ratio per year for military personnel)
Unit: 1/year

Description: Military service period is about 8 years (see our estimation for the parameter *Annual separation ratio* (item one in Table B). If in general people start their military service sometime between 18-25 years old and finish sometime between 25-32 years old, we can estimate the chance of death in this population from the civilians based on the data from USA Social Security Administration. The average chance of death in a window of eight-year life in those young periods is about 1.0%. The number of fatality during service in the military is relatively very low. For example, total of 6,607 in Iraq and Afghanistan were killed [23], the annual death of 0.02% for the whole military population.

Value: 0.010

Source: Authors' estimation from the data about life expectancy in the US using USA Social Security Administration [22].

Section 3: Model Formulation

We list the model formulation in the same format that appears in Vensim software (see the Vensim model, *PTSD_Simulation.mdl*, in the supplementary files). Model equations are listed for (a) simulation set-up, (b) model's main equations, and (c) experimental set-up.

3.1 Simulation set-up

1. INITIAL TIME = 2000
   Units: year

2. FINAL TIME = 2025
   Units: year

3. TIME STEP = 0.25
   Units: year

4. SAVEPER = TIME STEP
   Units: year

3.2 Model's main equations

5. "Healthy Military (PMH)"=
   INTEG (Recruitment of healthy-Developing illness in CZ-Developing illness not in CZ -PMH death rate-Rate of healthy separation, Initial PMH)

   Units: persons

6. "Healthy Veterans (PVH)"=
   INTEG (Rate of healthy separation+ Return of healthy veterans with PTSD history-Death rate of PVH, Initial PVH)

   Units: persons

7. "PM,ID: Ill-Diagnosed Military NotDeployed"=
   INTEG (Rate of diagnosis during service NotDeployed- PMIDn death rate-Separation of NotDeployed with PTSD -"Quitting treatment rate for not-deployed military", "initial PM,ID NotDeployed")

   Units: persons

8. "PM,ID: Ill-Diagnosed Military OEF & OIF"=
   INTEG ("Rate of diagnosis during service OEF & OIF"-PMID death rate-"Separation of OEF & OIF with PTSD"-"Quitting treatment rate for OEF & OIF in military", "initial PMID OEF&OIF")

   Units: persons

9. "PM,IU: Ill-Undiagnosed Military NotDeployed"=
INTEG (Rate of developing illness NotDeployed+ Recruitment of unhealthy- PMIUn death ratio -Rate of diagnosis during service NotDeployed- Separation of NotDeployed with unknown illness, initial PMU NotDeployed)

Units: persons

10. "PM,IU: Ill-Undiagnosed Military OEF & OIF"=
INTEG ("Developing illness OEF&OIF"-PMIU death rate-"Rate of diagnosis during service OEF & OIF"-"Separation of OEF & OIF with unknown illness", "initial PMIU OEF&OIF")

Units: persons

11. "PV,ID: Ill-Diagnosed Veterans of Pre-2000"=
INTEG ("Rate of diagnosis after service of Pre-2000"-"Death rate of PV,ID of Pre-2000"-"Quitting treatment rate for Pre-2000", "initial PVID Pre-2000")

Units: persons

12. "PV,ID: Veterans NotDeployed"=
INTEG (Separation of NotDeployed with PTSD+ Rate of diagnosis after service NotDeployed -"Quitting treatment rate for not-deployed veterans"-"Death rate of PV,ID of NotDeployed", "initial PV,ID NotDeployed")

Units: persons

13. "PV,ID: Veterans of OEF & OIF"=
INTEG ("Rate of diagnosis after service OEF & OIF"+ "Separation of OEF & OIF with PTSD"-"Death rate of PV,ID of OEF&OIF"-"Quitting treatment rate for OEF & OIF veterans", "initial PVID OEF&OIF")

Units: persons

14. "PV,IU: Ill-Undiagnosed Veterans NotDeployed"=
INTEG (Separation of NotDeployed with unknown illness-"Death rate of PV,IU of Not deployed"-Rate of diagnosis after service NotDeployed+ Separation not deployed with Ineffective treatment, initial VIU NotDeployed)

Units: persons

15. "PV,IU: Ill-Undiagnosed Veterans OEF & OIF"=
INTEG "Separation of OEF & OIF with unknown illness"-"Death rate of PV,IU of OEF&OIF"-"Rate of diagnosis after service OEF & OIF"+ "Separation OEF & OIF with Ineffective treatment", "initial PVIU OEF&OIF")

Units: persons

16. "PV,IU: Ill-Undiagnosed Veterans of Pre-2000"=
INTEG (-"Death rate of PV,IU of Pre-2000"-"Rate of diagnosis after service of Pre-2000", "initial VAU Pre-2000")

Units: persons

17. "PM,ID: Ill-Diagnosed Military"=
"PM,ID: Ill-Diagnosed Military NotDeployed"+"PM,ID: Ill-Diagnosed Military OEF & OIF"

Units: persons

18. "initial PM,ID NotDeployed"=
Initial rate of diagnosis of nondeployed/(Quitting treatment ratio in Military+ Separation ratio of people who have had PTSD)

Units: persons

19. initial PMU NotDeployed=
"initial PM,ID NotDeployed"*(Separation ratio of people who have had PTSD+ Quitting treatment ratio in Military)/Ratio revealing symptoms military

Units: persons

20. Initial PMH=
Initial military population - ("initial PMIU OEF&OIF"+ "initial PMID OEF&OIF"+ initial PMU NotDeployed +"initial PM,ID NotDeployed")

Units: persons

21. Initial PVH=
initial Veterans population-"initial PVID OEF&OIF"-"initial PVIU OEF&OIF"

-initial VIU NotDeployed-"initial VAU Pre-2000"-"initial PVID Pre-2000"-"initial PV,ID NotDeployed"

Units: persons

22. Post Treatment Stage Military NotDeployed=
INTEG ("Quitting treatment rate for not-deployed military"-"Separation not-deployed veterans with unknown status but history of PTSD",0)

Units: persons

23. "Post Treatment Stage Military OEF & OIF"=
INTEG ("Quitting treatment rate for OEF & OIF in military"-"Separation OEF & OIF veterans with unknown status but history of PTSD", 0)

Units: persons

24. "Rate of diagnosis during service OEF & OIF"=
"PM,IU: Ill-Undiagnosed Military OEF & OIF"*Ratio revealing symptoms military

Units: persons/year

25. Rate of diagnosis during service NotDeployed=
Min("PM,IU: Ill-Undiagnosed Military NotDeployed"/Min time to screen, A*"Rate of diagnosis during service OEF & OIF"+ B)

Units: persons/year

26. "(3) Rate of diagnosis during service"=
Rate of diagnosis during service NotDeployed+"Rate of diagnosis during service OEF & OIF"

Units: persons/year

27. Diagnosis multiplier=
(K*"PM,ID: Ill-Diagnosed Military")^Alpha

Units: Dmnl

28. Ratio revealing symptoms for pre2000 veterans=
Diagnosis multiplier*"Ratio revealing symptoms post-military"

Units: 1/year

29. "Rate of diagnosis after service of Pre-2000"=
"PV,IU: Ill-Undiagnosed Veterans of Pre-2000"*Ratio revealing symptoms for pre2000 veterans

Units: persons/year

30. "Rate of diagnosis after service OEF & OIF"=
"PV,IU: Ill-Undiagnosed Veterans OEF & OIF"*"Ratio revealing symptoms post-military"

Units: persons/year

31. Rate of diagnosis after service NotDeployed=
"PV,IU: Ill-Undiagnosed Veterans NotDeployed"*"Ratio revealing symptoms post-military"

Units: persons/year

32. "(8) Rate of diagnosis after service"=
"Rate of diagnosis after service of Pre-2000"+"Rate of diagnosis after service OEF & OIF"+ Rate of diagnosis after service NotDeployed

Units: persons/year

33. "Death rate of PV,ID of NotDeployed"=
"Death ratio for PV,ID NotDeployed"*"PV,ID: Veterans NotDeployed"

Units: persons/year

34. "Death rate of PV,ID of OEF&OIF"=
"Death ratio for PV,ID of Veterans of OEF & OIF"*"PV,ID: Veterans of OEF & OIF"

Units: persons/year

35. "Death rate of PV,ID of Pre-2000"=
"Death ratio for PV,ID Pre-2000"*"PV,ID: Ill-Diagnosed Veterans of Pre-2000"

Units: persons/year

36. "Death rate of PV,IU of Not-deployed"=
"Death ratio for PV, IU NotDeployed"*"PV,IU: Ill-Undiagnosed Veterans NotDeployed"

Units: persons/year

37. "Death rate of PV, IU of OEF&OIF"=
"Death ratio for PV, IU of Veterans of OEF & OIF"*"PV, IU: Ill-Undiagnosed Veterans OEF & OIF"

Units: 1/year

38. "Death rate of PV,IU of Pre-2000"=
"Death ratio for PV, IU Pre-2000"*"PV,IU: Ill-Undiagnosed Veterans of Pre-2000"

Units: 1/year

39. Death rate of PVH=
Death ratio for PVH*"Healthy Veterans (PVH)"

Units: 1/year

40. PMH death rate=
Death ratio in military*"Healthy Military (PMH)"

Units: persons/year

41. PMID death rate=
Death ratio in military*"PM,ID: Ill-Diagnosed Military OEF & OIF"

Units: persons/year

42. PMIU death rate=
Death ratio in military*"PM,IU: Ill-Undiagnosed Military OEF & OIF"

Units: persons/year

43. PMIUn death ratio=
Death ratio in military*"PM,IU: Ill-Undiagnosed Military NotDeployed"

Units: persons/year

44. PMIDn death rate=
Death ratio in military*"PM,ID: Ill-Diagnosed Military NotDeployed"

Units: persons/year

45. total death rate in military=
PMH death rate+ PMID death rate+ PMIU death rate+ PMIUn death ratio+ PMIDn death rate

Units: persons/year

46. PMH in CZ=
"Healthy Military (PMH)"*"Ratio deployed to combat zone (CZ)"

Units: (Trauma/persons)/year

47. Trauma experienced by PMH=
PMH in CZ*Trauma rate per year per person in CZ

Units: Trauma/year

48. Chance of developing PTSD given CZ trauma=
Normal chance of developing PTSD in CZ/Resiliency effect

Units: persons/Trauma

49. Chance of developing PTSD in NON CZ=
Normal chance of developing PTSD in NON CZ/Resiliency effect

Units: 1/year

50. Developing illness in CZ=
Trauma experienced by PMH*Chance of developing PTSD given CZ trauma

Units: persons/year

51. PMH not in CZ=
"Healthy Military (PMH)"*(1-"Ratio deployed to combat zone (CZ)")

Units: persons

52. Developing illness not in CZ=
Chance of developing PTSD in NON CZ*PMH not in CZ

Units: persons/year

53. "Developing illness OEF&OIF"=
Developing illness in CZ

Units: 1/year

54. Separation of NotDeployed with unknown illness=
"PM,IU: Ill-Undiagnosed Military NotDeployed"*Separation ratio of ill undiagnosed service members

Units: persons/year

55. "Separation of OEF & OIF with unknown illness"=
"PM,IU: Ill-Undiagnosed Military OEF & OIF"*Separation ratio of ill undiagnosed service members

Units: persons/year

56. Separation of NotDeployed with PTSD=
Separation ratio of people who have had PTSD*"PM,ID: Ill-Diagnosed Military NotDeployed"

Units: persons/year

57. "Separation of OEF & OIF with PTSD"=
Separation ratio of people who have had PTSD*"PM,ID: Ill-Diagnosed Military OEF & OIF"

Units: persons/year

58. Ill exits from military=
Separation of NotDeployed with unknown illness +"Separation of OEF & OIF with unknown illness" +Separation of NotDeployed with PTSD +"Separation of OEF & OIF with PTSD"

Units: 1/year

59. "Separation not-deployed veterans with unknown status but history of PTSD" =
Post Treatment Stage Military NotDeployed*Separation ratio with history of PTSD

Units: persons/year

60. "Separation OEF & OIF veterans with unknown status but history of PTSD" =
"Post Treatment Stage Military OEF & OIF"*Separation ratio with history of PTSD

Units: persons/year

61. Separation with histroy of PTSD but current unknown status=
"Separation not-deployed veterans with unknown status but history of PTSD" +"Separation OEF & OIF veterans with unknown status but history of PTSD"

Units: persons/year

62. "Estimate of rate of healthy separation for pre-2014"=
Max (0,DELAY1i(exit data(Time),data adjustment delay,Initial rate of healthy separation)-Ill exits from military-Separation with histroy of PTSD but current unknown status- total death rate in military)

Units: persons/year

Comment: The data reports final year death numbers. With a half a year delay we assume death is uniformly distributed over the year.

63. "PM,IU: Ill-Undiagnosed Military"=
"PM,IU: Ill-Undiagnosed Military NotDeployed"+"PM,IU: Ill-Undiagnosed Military OEF & OIF"

Units: persons

64. Service members with PTSD=
"PM,ID: Ill-Diagnosed Military" +"PM,IU: Ill-Undiagnosed Military"

Units: persons

65. Service members with history of PTSD and current unknown status=
Post Treatment Stage Military NotDeployed+"Post Treatment Stage Military OEF & OIF"

Units: persons

66. Total number of service members=
"Healthy Military (PMH)"+Service members with PTSD+ Service members with history of PTSD and current unknown status

Units: persons

67. Rate of healthy separation=
IF THEN ELSE(Time>2014 , "Estimate of rate of healthy separation for post-2014", "Estimate of rate of healthy separation for pre-2014")

Units: persons/year

68. Total exit from military=
Rate of healthy separation+ Ill exits from military+ Separation with history of PTSD but current unknown status+ total death rate in military

Units: persons/year

69. "Estimate of rate of recruitment for post-2014"=
max(0,Military size gap/Time to fill the gap+ Total exit from military)

Units: persons/year

70. Healthy separation not deployed with history of PTSD=
ratio of effective treatment*"Separation not-deployed veterans with unknown status but history of PTSD"

Units: persons/year

71. "Healthy separation OEF & OIF with history of PTSD"=
ratio of effective treatment*"Separation OEF & OIF veterans with unknown status but history of PTSD"

Units: persons/year

72. PTSD Cost for Diagnosed Military NotDeployed=
"PM,ID: Ill-Diagnosed Military NotDeployed"*PTSD Cost per Diagnosed Military(Time)

Units: dollar

73. “PTSD Cost for Diagnosed Military OEF & OIF"=
"PM,ID: Ill-Diagnosed Military OEF & OIF"*PTSD Cost per Diagnosed Military (Time)

Units: dollar

74. PTSD Cost for Diagnosed Veterans NotDeployed=
PTSD Cost per Diagnosed Veterans (Time)*"PV,ID: Veterans NotDeployed"

Units: dollar

75. "PTSD Cost for Diagnosed Veterans OEF & OIF"=
PTSD Cost per Diagnosed Veterans (Time)*"PV,ID: Veterans of OEF & OIF"

Units: dollar

76. "PTSD Cost for Diagnosed Veterans Pre-2000"=
PTSD Cost per Diagnosed Veterans (Time)*"PV,ID: Ill-Diagnosed Veterans of Pre-2000"

Units: dollar

77. PTSD Cost in Military=
"PM,ID: Ill-Diagnosed Military"*PTSD Cost per Diagnosed Military (Time)

Units: dollar

78. "PV,ID: Ill-Diagnosed Veterans"=
"PV,ID: Ill-Diagnosed Veterans of Pre-2000"+"PV,ID: Veterans of OEF & OIF"+"PV,ID: Veterans NotDeployed"

Units: persons

79. PTSD Cost in VA=
PTSD Cost per Diagnosed Veterans (Time)*"PV,ID: Ill-Diagnosed Veterans"

Units: dollar

80. "PV,IU: Ill-Undiagnosed Veterans"=
"PV,IU: Ill-Undiagnosed Veterans of Pre-2000"+"PV,IU: Ill-Undiagnosed Veterans OEF & OIF"+ "PV,IU: Ill-Undiagnosed Veterans NotDeployed"

Units: persons

81. Veterans with PTSD=
"PV,ID: Ill-Diagnosed Veterans"+"PV,IU: Ill-Undiagnosed Veterans"

Units: persons

82. Total number of veterans=
"Healthy Veterans (PVH)"+Veterans with PTSD

Units: persons

83. PTSD prevalence among Veterans=
Veterans with PTSD/Total number of veterans

Units: Dmnl

84. PTSD prevalence in Military=
Service members with PTSD/Total number of service members

Units: Dmnl

85. "Quitting treatment rate for not-deployed military"=
Quitting treatment ratio in Military*"PM,ID: Ill-Diagnosed Military NotDeployed"

Units: 1/year

86. "Quitting treatment rate for not-deployed veterans"=
Quitting treatment ratio for veterans*"PV,ID: Veterans NotDeployed"

Units: persons/year

87. "Quitting treatment rate for OEF & OIF in military"=
Quitting treatment ratio in Military*"PM,ID: Ill-Diagnosed Military OEF & OIF"

Units: persons/year

88. "Quitting treatment rate for OEF & OIF veterans"=
Quitting treatment ratio for veterans*"PV,ID: Veterans of OEF & OIF"

Units: persons/year

89. "Quitting treatment rate for Pre-2000"=
Quitting treatment ratio for veterans*"PV,ID: Ill-Diagnosed Veterans of Pre-2000"

Units: persons/year

90. Rate of developing illness NotDeployed=
Developing illness not in CZ

Units: persons/year

91. Recruitment of healthy=
(1-Unealthy recruitment ratio)*Recruitment

Units: persons/year

92. Recruitment of unhealthy=
Unhealthy recruitment ratio*Recruitment

Units: persons/year

93. Separation of healthy Veterans with history of PTSD=
"Healthy separation OEF & OIF with history of PTSD"+ Healthy separation not deployed with history of PTSD

Units: persons/year

94. Return of healthy veterans with PTSD history=
Separation of healthy Veterans with history of PTSD+ "Quitting treatment rate for OEF & OIF veterans"

Units: persons/year

95. Separation not deployed with Ineffective treatment=
(1-ratio of effective treatment)*"Separation not-deployed veterans with unknown status but history of PTSD"

Units: persons/year

96. "Separation OEF & OIF with Ineffective treatment"=
(1-ratio of effective treatment)*"Separation OEF & OIF veterans with unknown status but history of PTSD"

Units: persons/year

97. Separation of Ill Undiagnosed Veterans with history of PTSD=
"Separation OEF & OIF with Ineffective treatment"+ Separation not deployed with Ineffective treatment

Units: persons/year

(c) Experimental set-up

98. Resiliency effect=
Normal Resiliency*max (0, 1+Smooth (step(Percent Change in Resiliency/100,Policy implementation time), Time to implement policy))

Units: Dmnl

99. Ratio revealing symptoms military=
Normal Ratio revealing symptoms military* max(0,1+Smooth(step(Percent Change in Screening/100,Policy implementation time),Time to implement policy))

Units: 1/year

100. Quitting treatment ratio in Military=
Normal quitting ratio in Military*max(0,1+Smooth(step(Percent Change in Treatment in Military/100,Policy implementation time),Time to implement policy))

Units: 1/year

101. Deployment to a hypothetical war=
Intensity of a hypothetical war in comparison to Iraq*Trauma multiplier in Iraq*"Deployment during Iraq war (data)"(Time-"Time for an Iraq-like war")

Units: Dmnl

102. Steady state deployment=
IF THEN ELSE (Time>2015, Intensity in steady state in comparison to Iraq*Deployment ratio in Iraq during the war*Trauma multiplier in Iraq,0)

Units: Dmnl

103. "Deployment to CZ-like areas post-2014"=
Deployment to a hypothetical war+ Steady state deployment

Units: persons

104. Desired number of service members=
Total service members in 2014

Units: Dmnl

105. "Ratio deployed to combat zone (CZ)"=
IF THEN ELSE(Time<=2015,"Deployment to CZ areas pre-2014 (OEF & OIF)" (Time),"Deployment to CZ-like areas post-2014")

Units: Dmnl

106. "Estimate of rate of healthy separation for post-2014"=
"Healthy Military (PMH)"*Separation ratio of healthy service members

Units: persons/year

107. "Estimate of rate of recruitment for post-2014"=
max(0,Military size gap/Time to fill the gap+Total exit from military)

Units: persons/year

108. Military size gap=
Desired number of service members-Total number of service members

Units: year

109. Recruitment=
IF THEN ELSE(Time>2014,"Estimate of rate of recruitment for post-2014","Recruitment data pre-2014"(Time))

Units: persons/year

Section 4: Model Calibration

In any modeling, the overall goal is to estimate as many parameters as possible directly from data. But sometimes there are some parameters for which data are not gathered, reliable data are not available, or we could not find them. In these situations, we can statistically calibrate the model to data by estimating the unknown parameters.

In simple words, calibration is about using statistical methods to estimate parameter values for which we do not have data. It is important to conduct sensitivity analysis for estimated parameters, examining the model's robustness to changes in the estimations.

We conduct partial model calibration/testing [25] for parameter estimation. In the partial model calibration, different pieces of the model are separately calibrated. This method is known to provide relatively robust estimates and decrease the chances of over-fitting the model. It also helps understand which parts of the model are better representative of the reality and which parts are producing more errors.

4.1. Unknown parameters

Table C presents estimated parameters through calibration as well as the respective calibration steps. Overall, we have conducted six different calibration procedures (calibration procedures 1-6) for the total of eight unknown parameters. Sensitivity of our results to parameter estimations is presented in Section 5.

Table C. Estimated (unknown) parameters in the model

No	Parameter	Value	Calibration step
1	Quitting treatment ratio in military	0.125	Calibration procedure 1
2	Quitting treatment ratio in VA	0.145	Calibration procedure 2
3	Ratio revealing symptoms in military	0.043	Calibration procedure 3
4	Ratio revealing symptoms for veterans	0.103	Calibration procedure 4
5	Effects of Iraq/Afghanistan wars on revealing symptoms for pre-2000		Calibration procedure 5
	$x = (K*P_{M,}ID)^{\alpha}$	K= 1.38E-06<br>α = 0.4959	
6	Rate of PTSD diagnosis for non-combat related reasons		Calibration procedure 6
	$y = y_c + a$*Rate of diagnosis OEF & OIF<br>y < PM,IU	y_c = 2267.8<br>a = 0.0936	

1. Quitting treatment ratio in military
   Unit: 1/year

   Description: Since there is no reliable data on effective treatment (Institute of Medicine, 2014), we focus on the number of people who stop receiving treatment. This ratio presents the portion of the military personnel who were receiving PTSD treatment in the previous time period (*t-1*), and are no longer under treatment (at time *t*). The degree of freedom (DOF) in this producer is one (described in more detail in calibration procedure 1), which means we have a highly accurate estimation.

   Source: Calibration procedure 1 (DOF=1)

   Value: 0.1347

2. Quitting treatment ratio in VA
   Unit: 1/year

   Description: Since there is no reliable data on effective treatment [1], we focus on the number of people who stop receiving treatment. This ratio is in fact veterans who were receiving PTSD treatment in VA facilities in the previous time period (*t-1*), and are no longer under treatment (at time *t*). Similar with the previous parameter, the DOF in this producer is one (described in more detail in calibration procedure 2), which means we have a highly accurate estimation.

   Source: Calibration procedure 2 (DOF=1).

   Value: 0.1457

3. Ratio revealing symptoms in military
   Unit: 1/year

   Description: Not all military members with PTSD show symptoms while serving in the military. There is no data on how many people have PTSD and are undiagnosed. Thus, we estimated the rate at which undiagnosed military personnel turn to diagnosed military personnel through calibration.

   Source: Calibration procedure 3 (DOF=1).

   Value: 0.0415

4. Ratio revealing symptoms post-military (Iraq and Afghanistan)
   Unit: 1/year

   Description: Many veterans who have PTSD gradually show their symptoms of illness. We estimated at rate at which undiagnosed veterans turn to diagnosed veterans through calibration.

   Source: Calibration procedure 4 (DOF=1)

   Value: 0.1001

5. Effects of Iraq/Afghanistan wars on revealing symptoms for pre-2000 veterans

   Unit: 1/year

   Description: Ratio of revealing symptoms for pre-2000 veterans is not necessarily the same as the ratio for more recent wars. Some veterans who served in the military before 2000 and developed PTSD may show symptoms of illness after 2000. Anecdotal evidence and the paper by Hermes, Rosenheck (10) suggest that the number of Vietnam veterans with PTSD is still increasing. One argument is that the war in Iraq and Afghanistan had triggered old memories exacerbating the situation of these veterans. This is one of the reasons we may see a growth in PTSD of pre-2000 veterans. Accordingly, we assumed:

   Ratio of revealing symptoms post-military for pre-2000 wars =

   *Ratio of revealing symptoms post-military * Effects of Iraq/Afgh wars =*

   Ratio of revealing symptoms post-military * $[K*P_{M,ID}{}^{\alpha}]$

   In this formulation, the term $[K*P_{M,ID}{}^{\alpha}]$ is used to represent the multiplier effect of recent wars. The logic is that with more PTSD cases from recent wars, we will hear proportionally more news about PTSD which affects new diagnosis in an increasing but declining slop.

   Source: Calibration procedure 5 (DOF=2)

   Values: K= 1.14E-3; α = 0.51

6. Rate of PTSD diagnosis for non-combat related reasons

   Unit: Persons/year

   Description: Ratio of revealing symptoms for people not deployed have been increasing. We presented this with a constant parameter (representing issues unrelated to wars) plus a linear term representing the growth trend. This growth trend is partially due to more screening and more attention to PTSD or hiring individuals with illness.

   Rate of PTSD diagnosis for non-combat related reasons =

   = y_c + *a*Rate of diagnosis OEF & OIF* + $b < P_{M, IU}$

   Source: Calibration procedure 6 (DOF=2)

   Values: y_c = 2267.8; a = 0.0936

4.2. Calibration procedures:

4.2.1 Calibration procedure 1:

- Input: *Rate of diagnosis [of PTSD] in Military* (Table A, item 8.1), *Separation ratio of ill-diagnosed* (Table B, item 1.3), and *fractional death rate for military personnel* (Table B, item 9).
- Payoff function: maximize the fit between the data and simulation of $P_{M,ID}$.
- Outcomes: the only unknown parameter, *Quitting treatment ratio in military* (Table C, item 1).
- DOF: 1

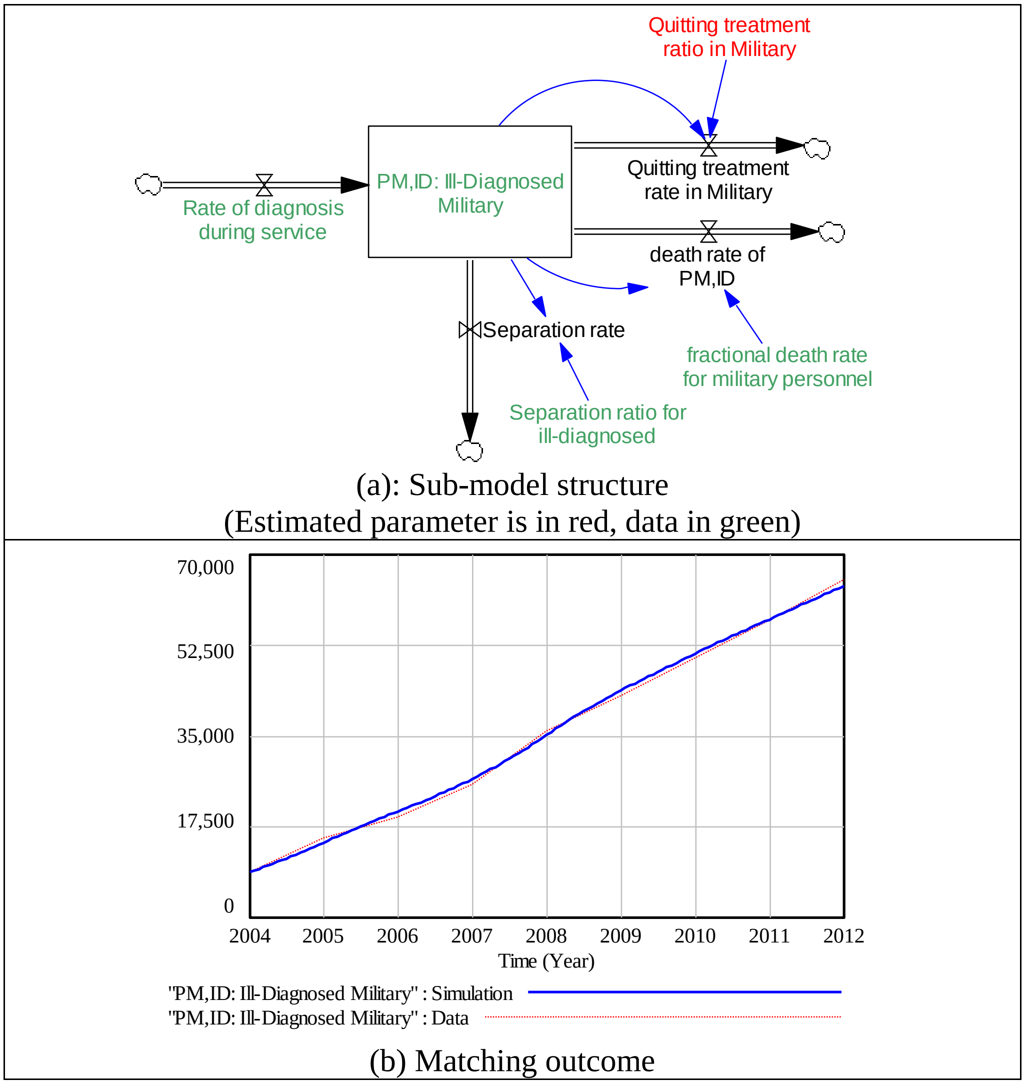

Figure G. Calibration procedure 1 including sub-model structure (a), and the matching outcome (b)

Calibration outcome: <u>*Quitting treatment ratio in military* = 0.125</u>

4.2.2 Calibration procedure 2:

- Input: *Rate of diagnosis in VA* (Table A, item 10), *fractional death rate for veterans* (Table B, item 8).
- Payoff function: maximize the fit between the data and simulation of $P_{V,ID}$.
- Outcomes: the only unknown parameter, *Quitting treatment ratio in VA* (Table C, item 2)
- DOF: 1

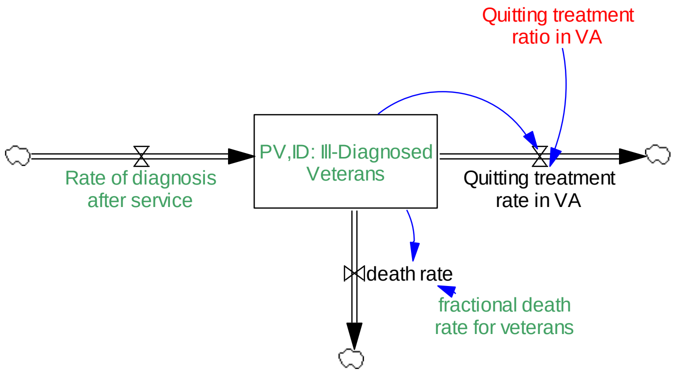

(a): Sub-model structure
(Estimated parameter is in red, data in green)

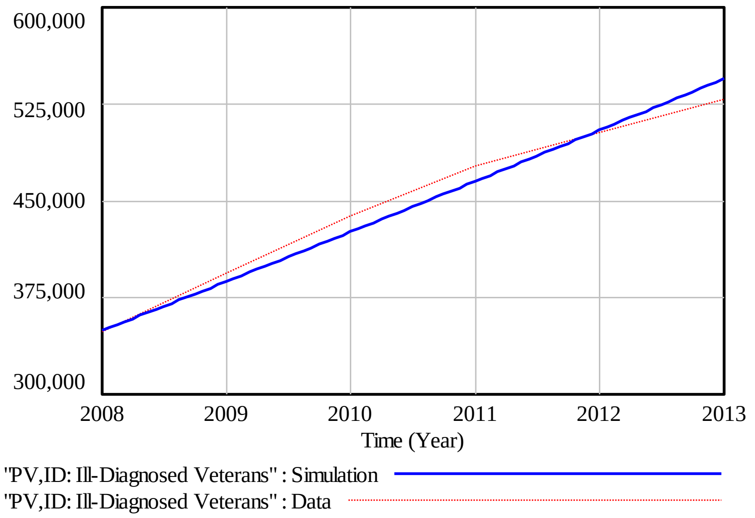

(b) Matching outcome

Figure H. Calibration procedure 2 including sub-model structure (a), and the matching outcome (b)

Calibration outcome: <u>*Quitting treatment ratio in VA* = 0.145</u>

4.2.3 Calibration procedure 3:

- Input: *Deployment to Combat Zone* (Table A, items 2 and 3), *Chance of developing PTSD given combat zone trauma*, *trauma rate per year per person in combat zone* (Table B, items 2 and 3), death ratio of PM,IU (assumed equal to *fractional death rate for military personnel*; Table B, item 9), and *Separation of ill undiagnosed service members* (Table B, item 1.1). Initial PMIU OEF & OIF is zero since simulation starts before these wars. PMH is calculated endogenously using recruitment rate and the military population (Table A, items 4 and 1).
- Payoff function: maximize the fit between the data and simulation of Diagnosis rate of military personnel served in OIF and OEF.
- Outcomes: the only unknown parameter, *Ratio revealing symptoms in military* (Table C, item 3)
- DOF: 1

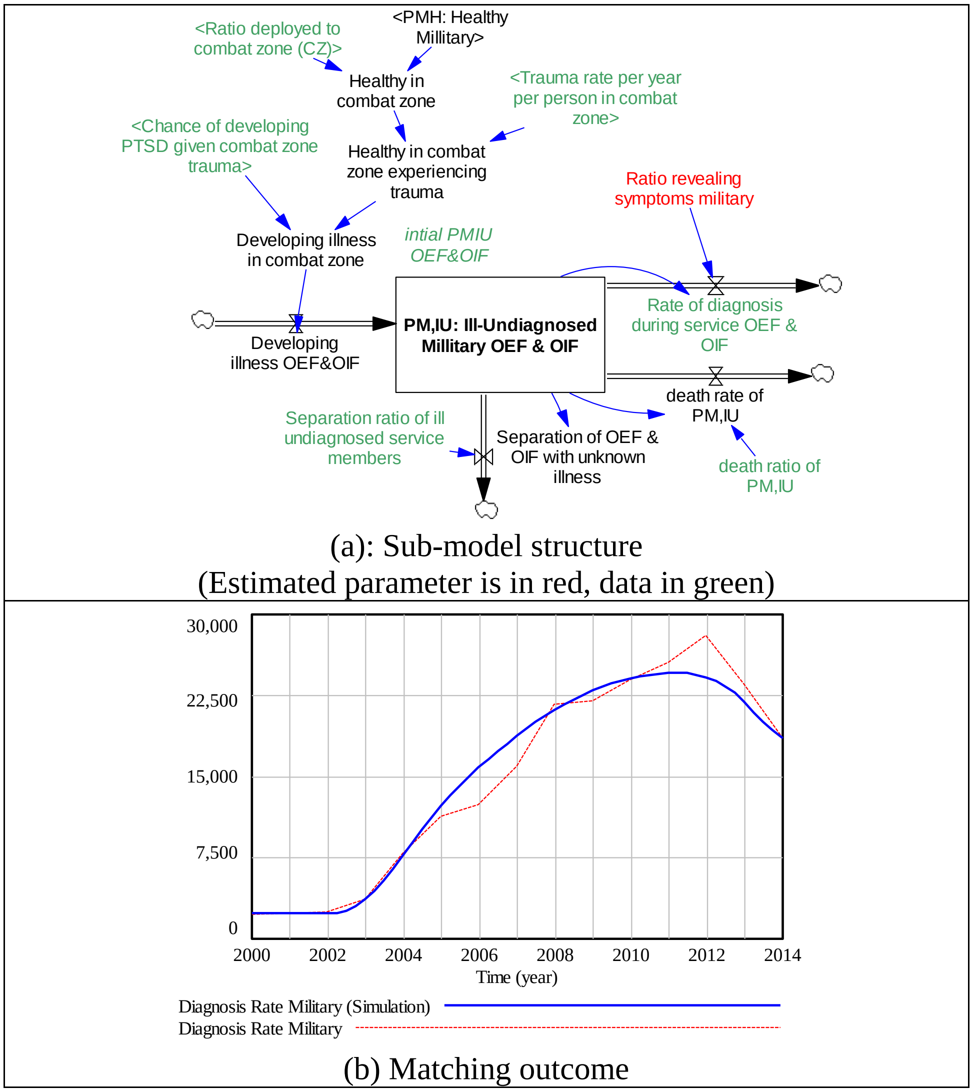

(a): Sub-model structure
(Estimated parameter is in red, data in green)

(b) Matching outcome

Figure I. Calibration procedure 3 including sub-model structure (a), and the matching outcome (b)

Calibration outcome: <u>*Ratio revealing symptoms in military* = 0.043</u>

4.2.4 Calibration procedure 4:

- Input: *Death ratios for PV,IU and PV,ID of Veterans of OEF and OIF* (assumed to be equal to *Fractional death rate for veterans*; Table B, item 8). In addition, Separation of ODF and OIF are calculated from the previous calibrated sub-structures (calibration procedure 3 and calibration procedure 1). Stopping treatment for veterans is the outcome of calibration procedure 2.
- Payoff function: maximize the fit between the data and simulation of OEF and OIF veterans diagnosed with PTSD.
- Outcomes: The only unknown parameter, *Ratio revealing symptoms* for Iraq/Afghanistan veterans (Table C, item 4).
- DOF: 1

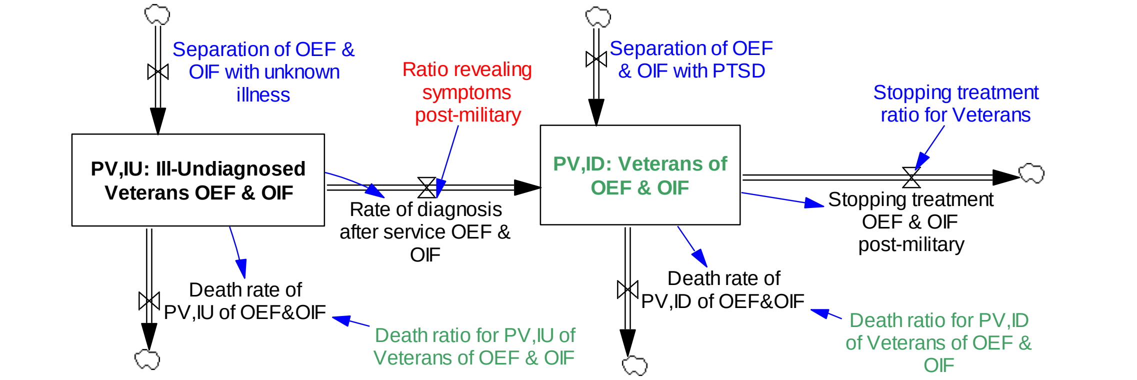

(a): Sub-model structure
(Estimated parameter is in red, data in green, the results of previous calibration procedures in blue)

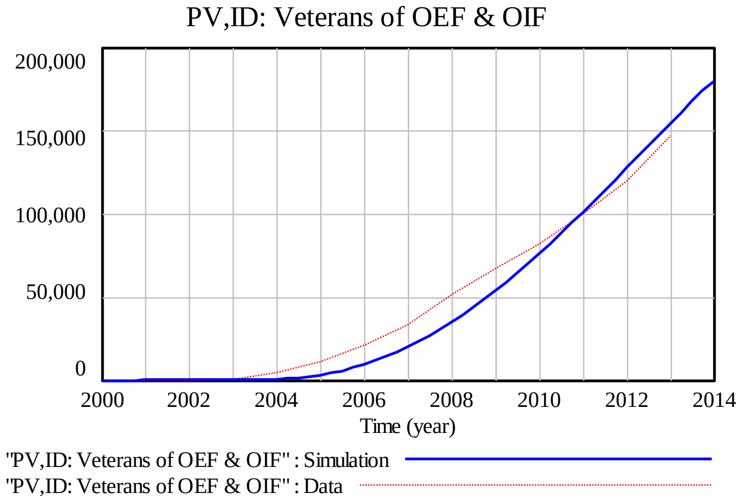

(b) Matching outcome

Figure J. Calibration procedure 4 including sub-model structure (a), and the matching outcome (b)

Calibration outcome: *Ratio revealing symptoms for Iraq/Afghanistan veterans* = 0.103

4.2.5 Calibration procedure 5:

- Input: PM,ID (*PTSD diagnosed in military*, Table A, item 7), *Death ratios for PV,IU and PV,ID of Veterans of pre-2000 wars* (assumed to be equal to *Fractional death rate for veterans*; Table B, item 8), *Initial PVID* (Table A, item 9.2). In addition, *ratio revealing symptoms post military* and *stopping treatment for veterans* is the outcomes of calibration procedures 2 and 4.
- Payoff function: maximize the fit between the data and simulation of PTSD Diagnosed veterans with pre-2000.
- Outcomes: Effects of Iraq/Afghanistan wars on revealing symptom for pre-2000 Veterans.
- Assumption: We first roughly estimated the initial value of VIU to limit potential values for k and α. Based on the population of the Vietnam war veterans and estimated ratios of PTSD, we set the initial value to be equal to 5 million.
- DOF: 1 (after making the assumption for the initial value).
- Note: We later conducted sensitivity analysis on the value of VIU. With 2DOF optimization, Vensim suggest the initial value to be equal to 5.4 million veterans which is very close to our initial assumption. The final results do not change when we change 5 to 5.4 million and even with ± 1 more million shift in this value.

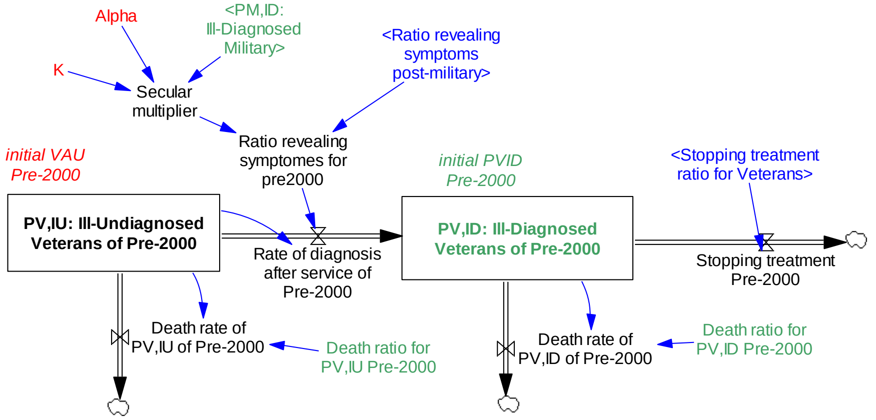

(a): Sub-model structure

(Estimated parameter is in red, data in green, the results of previous calibration procedures in blue)

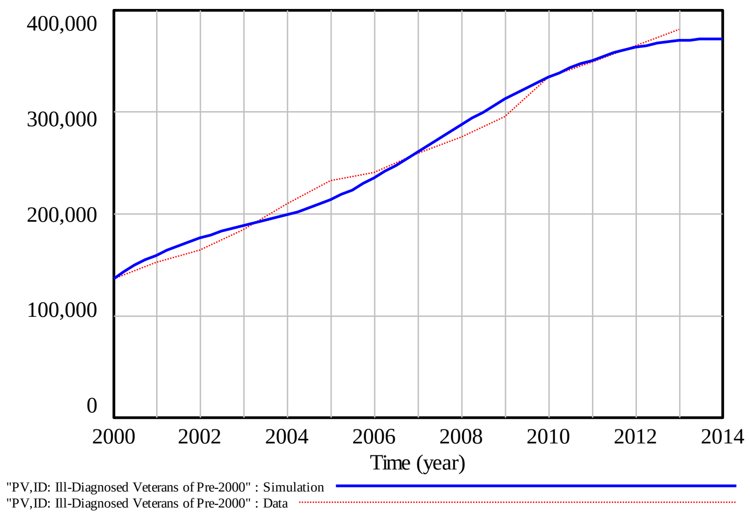

(b) Matching outcome

Figure K. Calibration procedure 5 including sub-model structure (a), and the matching outcome (b)

Calibration outcomes: <u>K= 1.38E-06 and α = 0.4959</u>

4.2.6 Calibration procedure 6:

- Input: Diagnosis rate of the military personnel deployed to Iraq and Afghanistan (Table A, item 8.1)
- Payoff function: maximize the fit between the data and simulation of Diagnosis rate of the military personnel who did not deploy.
- Outcomes: parameters to estimate diagnosis rate of non-combat related PTSD
- Assumption:

  $y = y_c + a$*Rate of diagnosis OEF & OIF $\quad < P_{M,IU}$
- DOF: 1

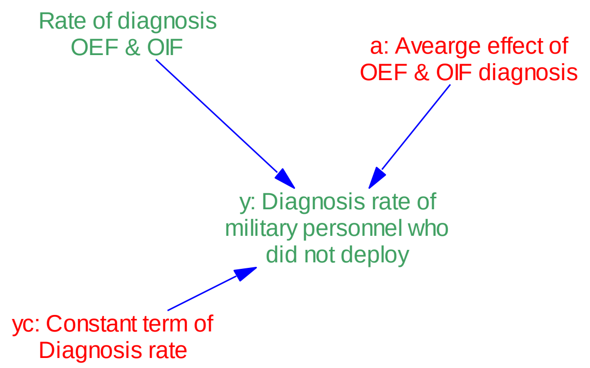

(a): Sub-model structure
(Estimated parameter is in red, data in green)

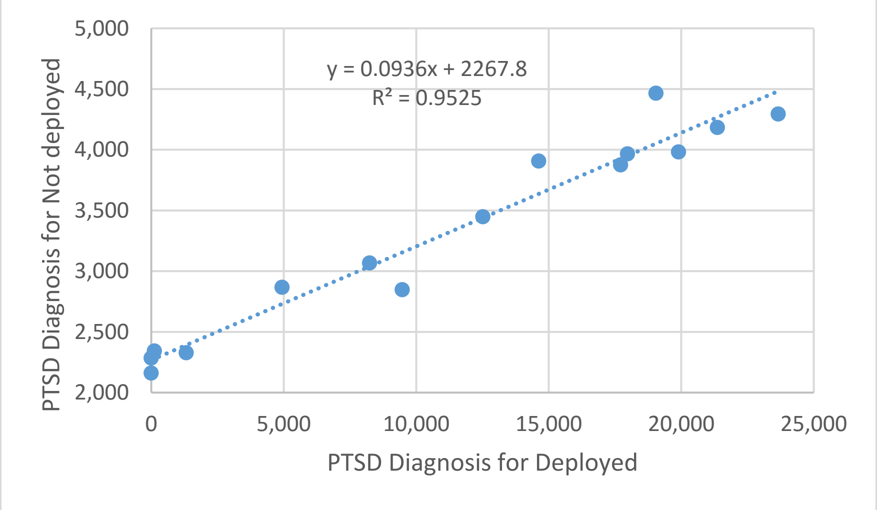

(b) Matching outcome

Figure L. Calibration procedure 6 including sub-model structure (a), and the matching outcome (b)

Calibration outcome: $y_c = 2267.8$ & $a = 0.0936$

Section 5: Model Validation and Sensitivity Analysis

We report the model's fidelity in replicating the data. Figure M shows the model outcomes in comparison with the data for variables: PTSD diagnosed cases in military, PTSD rate of diagnosis in military during service in Iraq or Afghanistan, PTSD rate of diagnosis in Military (non-deployed military personnel), PTSD diagnosed cases in VA, PTSD diagnosed cases in VA (pre-2000), and PTSD diagnosed cases in VA (post-2000 wars, Iraq and Afghanistan).

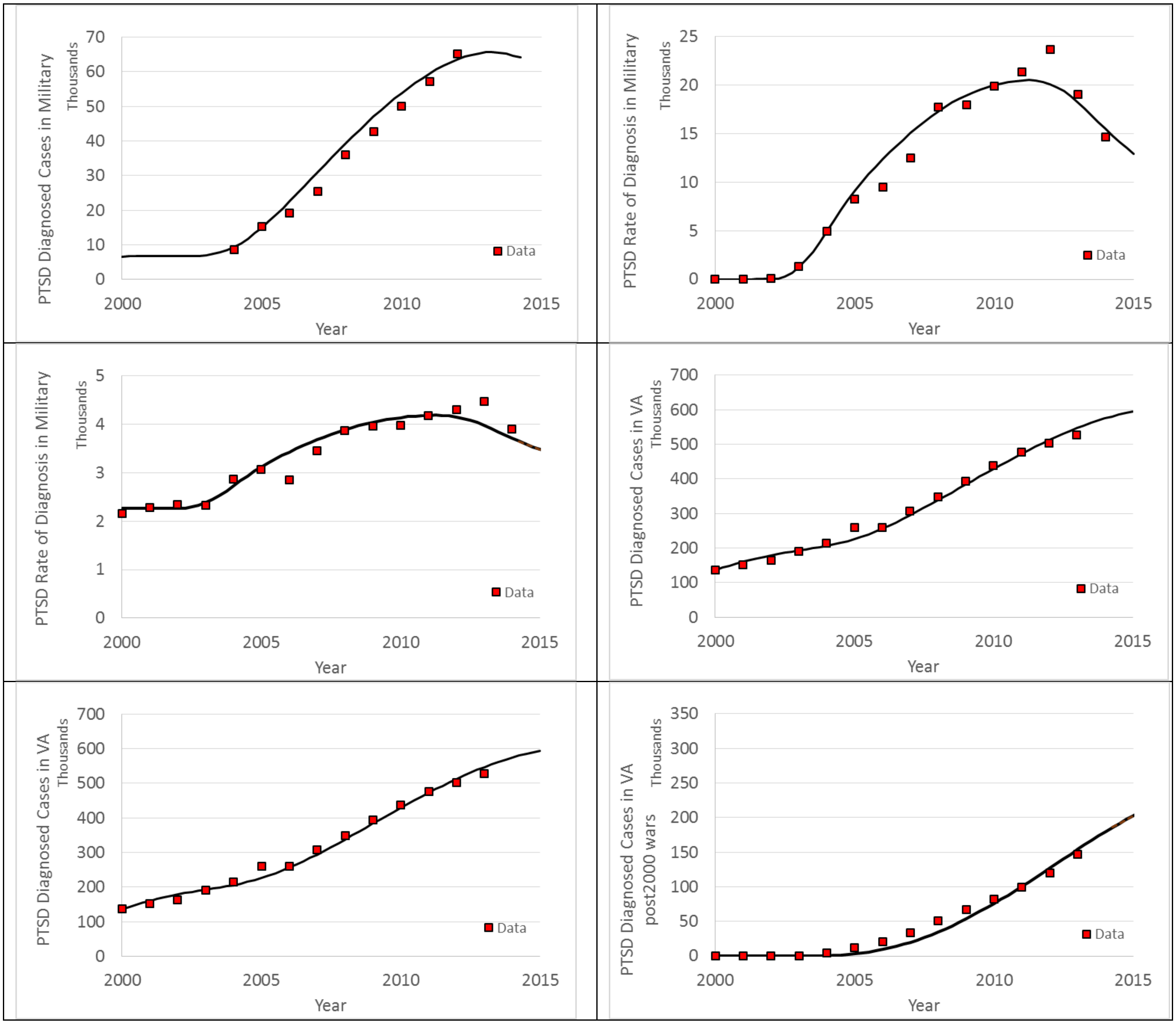

Figure M. Replication of the data

The model replicated the data with a high correlation. This is not a surprise since our model mainly focuses on the physics of the system, which is the flow of people. What technically validates the model is the simple logical flow in the model—the logic behind the inflows and outflows of the stock variables in the model, see the Vensim model, *PTSD_Simulation.mdl*, in the supplementary files).

We acknowledge the limitations of building complex models like the one we developed. To ensure the reliability of the model's outputs, we conduct sensitivity analysis on the estimated parameters and the ones that we doubt about (given the uncertainty in the data). Here we demonstrate change the value of those parameters (by ±50%) in our second scenario (2% deployment to intense/combat zones, see the article), and examine how the model outputs (PTSD prevalence in military and VA) vary. The results are qualitatively similar for scenarios 1 and 3, or anything between. Results are provided in Table D. We also include sensitivity results for changing all parameters at a time in the ranges of ±25% and ±50%, reported in Table E.

Table D. Sensitivity analysis results

No.	Parameter	Input	Outputs		Comments
		**Change in parameter**	**Change in military PTSD Prevalence (Y1)**	**Change in veterans PTSD Prevalence (Y2)**	Change in the parameter results in…
1	Separation ratio of ill-diagnosed	±50%	10% - 11%	10% - 10%	±0.5 percent point change in Y1; 0 percent point change in Y2.
2	Average number of traumas a deployed person experiences per year (traumas/year/person)	±50%	7% - 14%	10%-10%	±3.5 percent point change in Y1; 0 percent point change in Y2.
3	Unhealthy recruitment ratio	[0%-5%]*	10% - 13%	10% - 10%	±1.5 percent point change in Y1; 0 percent point change in Y2.
4	Chance of developing PTSD for non-combat related reasons	±50%	10% - 10%	10% - 10%	0 percent point change in Y1; 0 percent point change in Y2.
5	Quitting treatment ratio in military	±50%	10% - 10%	10% - 10%	0 percent point change in Y1; 0 percent point change in Y2.

6	Quitting treatment ratio in VA	±50%	10% - 10%	9% - 11%	0 percent point change in Y1;<br>±1 percent point change in Y2.
7	Ratio revealing symptoms in military	±50%	10% - 10%	10% - 10%	0 percent point change in Y1;<br>0 percent point change in Y2.
8	Ratio revealing symptoms for veterans	±50%	10% - 10%	9% - 11%	0 percent point change in Y1;<br>±1 percent point change in Y2.

* In the base run, the model assumes 1% unhealthy recruitment (people have a history of PTSD). For this parameter, we test a much wider range, due to some speculations about the correct value of this parameter.

Simulation results are also reported in Figure N, showing 50%, 75%, 95%, and 100% intervals based on 200 times Monte Carlo simulations.

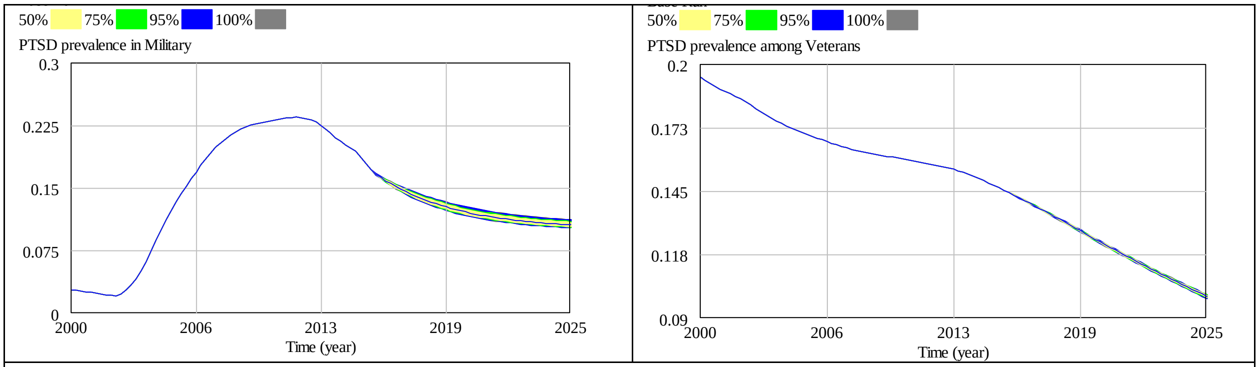

(a) Sensitivity of PTSD prevalence in military and VA to ±50% change in parameter "Separation ratio of ill-diagnosed"

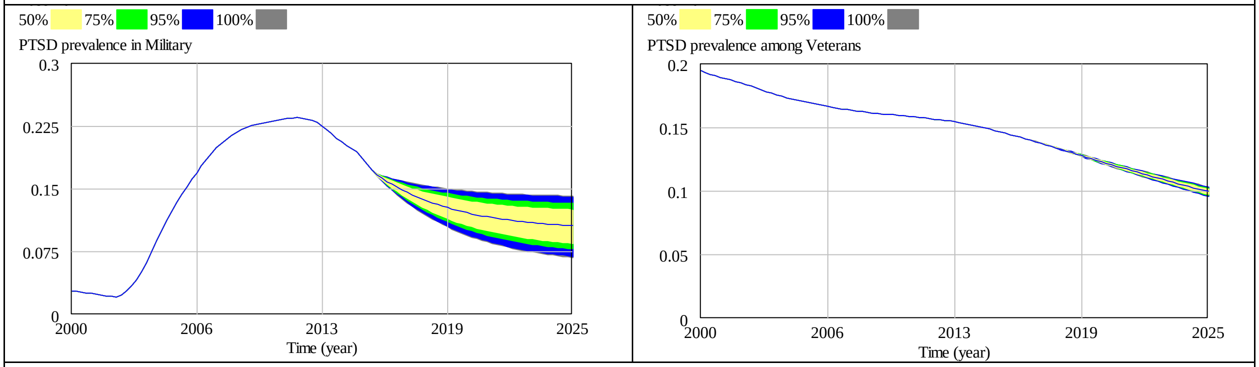

(b) Sensitivity of PTSD prevalence in military and VA to ±50% change in parameter "Average number of traumas a deployed person experiences per year"

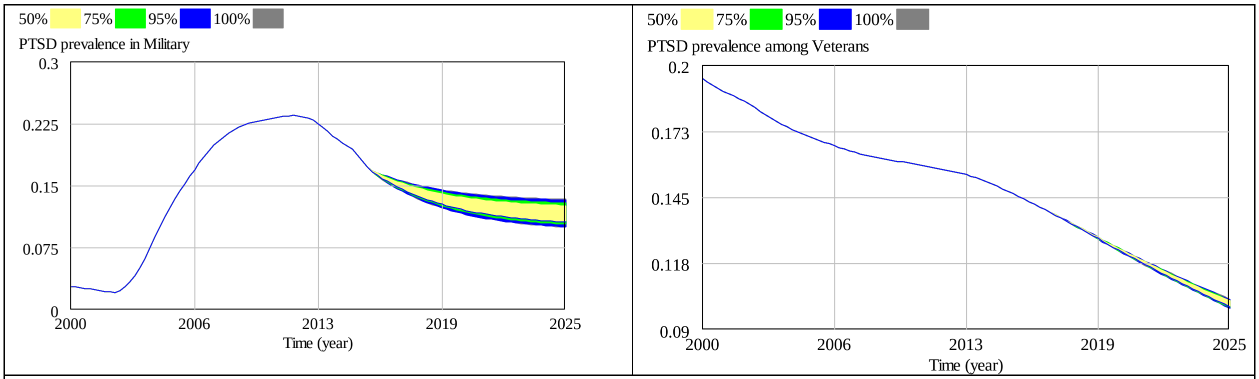

(c) Sensitivity of PTSD prevalence in military and VA to ±500% change in parameter “Unhealthy recruitment ratio”

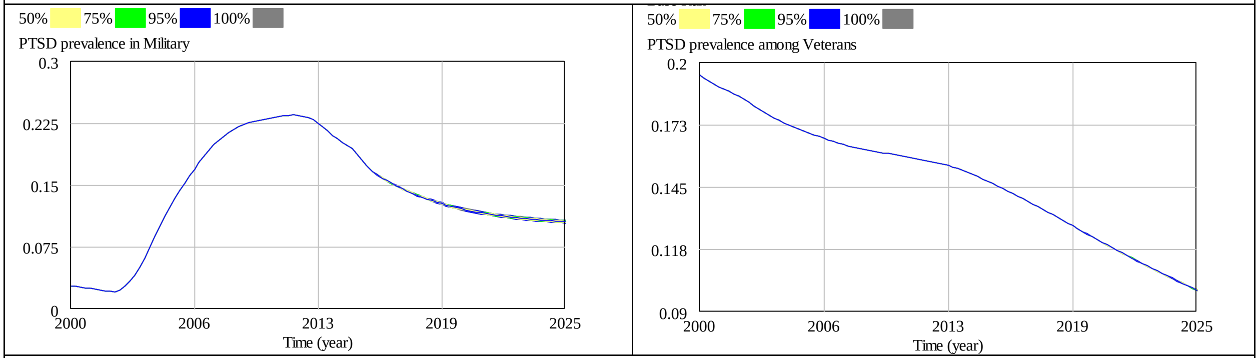

(d) Sensitivity of PTSD prevalence in military and VA to ±50% change in parameter “Chance of developing PTSD for non-combat related reasons”

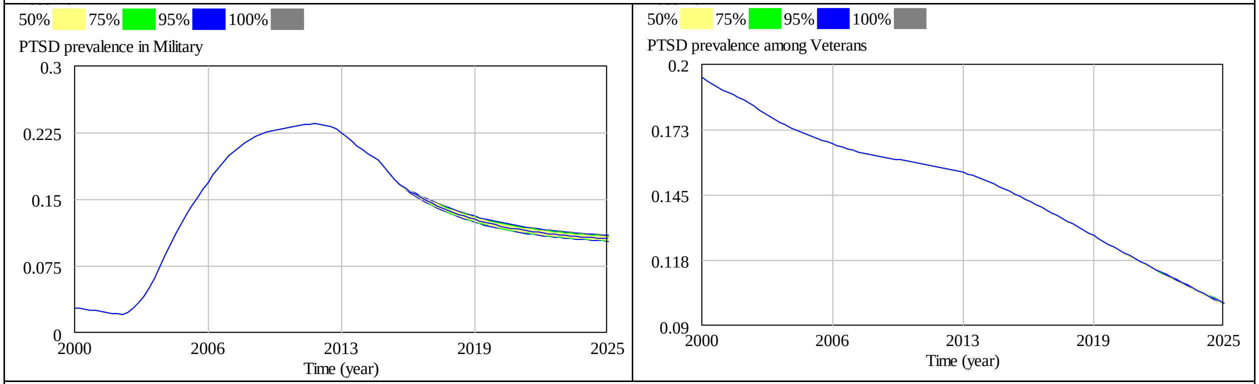

(e) Sensitivity of PTSD prevalence in military and VA to ±50% change in parameter “Quitting treatment ratio in military”

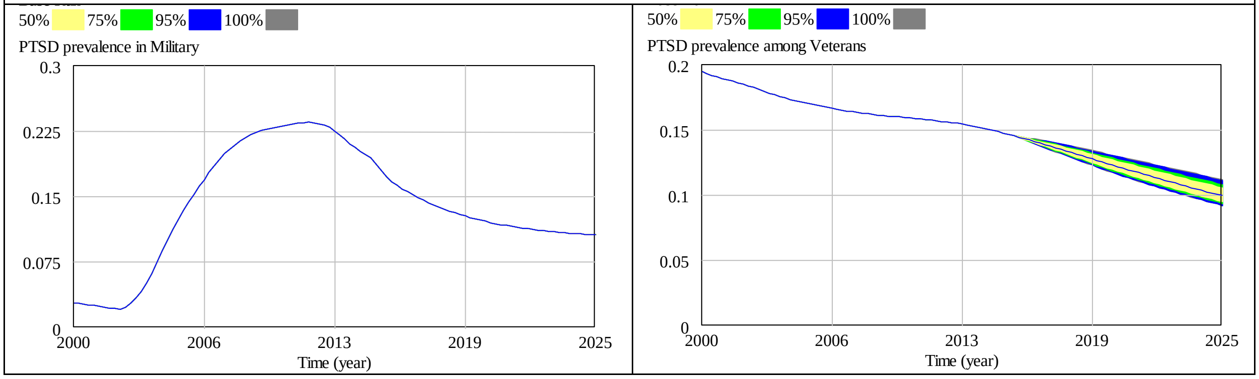

(f) Sensitivity of PTSD prevalence in military and VA to ±50% change in parameter "Quitting treatment ratio in VA"

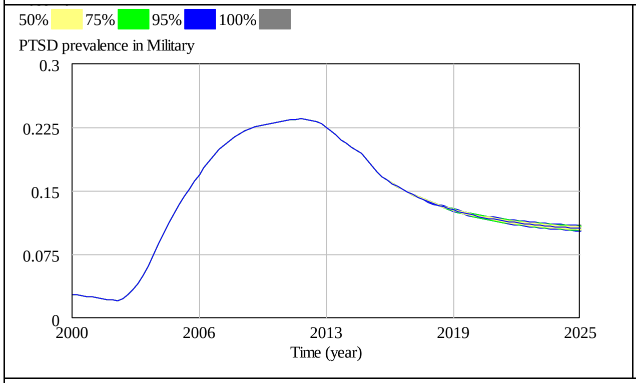

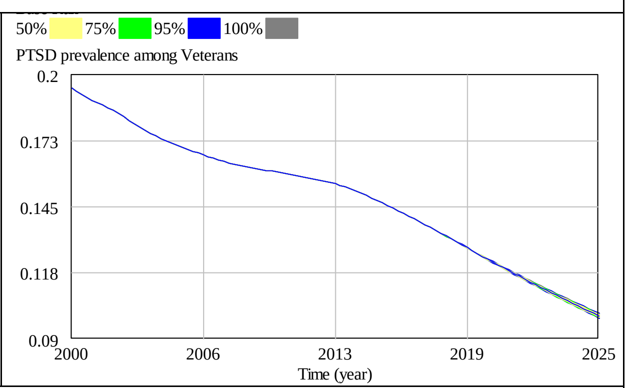

(g) Sensitivity of PTSD prevalence in military and VA to ±50% change in parameter "Ratio revealing symptoms in military"

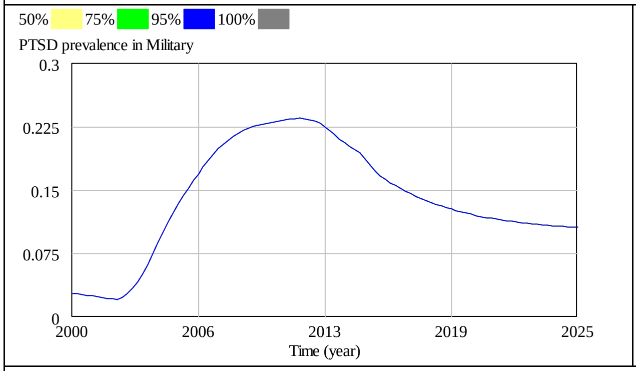

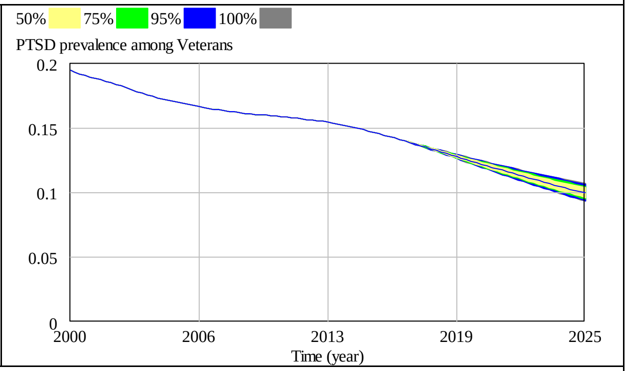

(h). Sensitivity of PTSD prevalence in military and VA to ±50% change in parameter "Ratio revealing symptoms for veterans"

Figure N. Sensitivity analysis results

In summary, the model outcomes are fairly robust to the changes in all parameters, except 1) Average number of traumas a deployed person experiences per year, and 2) Unhealthy recruitment ratio. For the first parameter, the implication is that if the remaining military personnel in Iraq or other places face with more trauma, e.g., due to escalating situations, there will be more PTSD incidence. This is not a surprise. Our model's prediction is specifically for 2% deployment in the current situation. And for the second parameter, Unhealthy recruitment ratio, the analysis presents that if less healthy people are hired, we will see a higher PTSD prevalence. This is also not a surprise. Our model runs based on the current status, but we acknowledge that if for any reason less healthy/resilient people are hired in the military in the future, PTSD prevalence will increase. However, we expect potential changes to influence the prevalence in the military by no more than 3 percent point.

Finally, we change all the parameters together to see the effects on the PTSD prevalence. The results are reported in Table E.

Table E. Sensitivity results due to changes in all 8 parameters in Table D

No.	Parameters	Inputs	Outputs		Comment
		Change in parameters	Change in military PTSD Prevalence (Y1)	Change in Veterans PTSD Prevalence (Y2)	Change in the parameter results in…
1	All 8 parameters in Table D	±25%	9%-14%	9%-11%	±2.5 percent point change in Y1; 1 percent point change in Y2.
2	All 8 parameters in Table D	±50%	7%-17%	8%-12%	±5 percent point change in Y1; ±2 percent point change in Y2.

The results present that the estimation of PTSD prevalence among veterans is reliable. However, for the military personnel it can change between 7% and 17% if all important parameters change by 50%. We would like to mention that the most sensitive parameters are the average number of traumas a deployed person experiences per year (traumas/year/person), and the unhealthy recruitment ratio in military. This is not a surprise as if both parameters change (e.g., the situation get more escalated in the middle east and we end up hiring less healthy military personnel), they may influence PTSD prevalence in the military.

References:

1. Institute of Medicine I. Washington (DC): National Academies Press (US)Copyright 2014 by the National Academy of Sciences. All rights reserved.; 2014.

2. D.o.D. Defense.gov News Release: DoD Announces Recruiting and Retention Numbers for Fiscal 2015, Through November 2014. 2015.

3. Liu J, Xiao Y, Li S, Liang W, Chen CP. Cyber security and privacy issues in smart grids. IEEE Communications Surveys & Tutorials. 2012;14(4):981-97.

4. Cronbach LJ. Coefficient alpha and the internal structure of tests. Psychometrika. 1951;16(3):297-334. doi: 10.1007/bf02310555.

5. D.o.D. 2013 DEMOGRAPHICS PROFILE OF THE MILITARY COMMUNITY. 2013.

6. Richardson C, Waldrop J. Veterans: Census 2000 Brief: U.S. CENSUS BUREAU; 2003 [cited 2015 7/11/2015]. Available from: https://www.census.gov/prod/2003pubs/c2kbr-22.pdf.

7. Bagalman E. The Number of Veterans That Use VA Health Care Services: A Fact Sheet 2014.

8. Fischer H. A Guide to U.S. Military Casualty Statistics: Operation Inherent Resolve, Operation New Dawn, Operation Iraqi Freedom, and Operation Enduring Freedom Congressional Research Service2014.

9. Rosenheck RA, Fontana AF. Recent Trends In VA Treatment Of Post-Traumatic Stress Disorder And Other Mental Disorders. Health Affairs. 2007. doi: 10.1377/hlthaff.26.6.1720.

10. Hermes ED, Rosenheck RA, Desai R, Fontana AF. Recent trends in the treatment of posttraumatic stress disorder and other mental disorders in the VHA. Psychiatric services. 2012;63(5):471-6. doi: 10.1176/appi.ps.201100432. PubMed PMID: 22422018.

11. D.o.D. DoD Announces Recruiting and Retention Numbers: Department of Defense; 2014 [cited 2015 7/9/2015]. Available from: http://www.defense.gov/releases.

12. DeFraites R, Vythilingam M. DoD Deployment Mental Health Assessments: A Review and Update. Falls Church, VA: Force Health Protection and Readiness. 2011.

13. Segal DR, Segal MW. America's military population: Population Reference Bureau Washington, DC; 2004.

14. Little JD. A proof for the queuing formula: L= λ W. Operations research. 1961;9(3):383-7.

15. 2013. Behavioral Health Task Force reports 'no systemic issues'.

16. Martin M. PTSD risk factors 2014 [cited 2015 7/15/2015]. Available from: http://www1.appstate.edu/~hillrw/PTSD%20MM/PTSD_riskfactors.html.

17. Hoge CW, Castro CA, Messer SC, McGurk D, Cotting DI, Koffman RL. Combat duty in Iraq and Afghanistan, mental health problems, and barriers to care. New England Journal of Medicine. 2004;351(1):13-22.

18. Monahan P, Hu Z, Rohrbeck P. Mental disorders and mental health problems among recruit trainees, US Armed Forces, 2000-2012. MSMR. 2013;20(7):13-8; discussion 6-8.

19. Kilpatrick DG, Resnick HS, Milanak ME, Miller MW, Keyes KM, Friedman MJ. National estimates of exposure to traumatic events and PTSD prevalence using DSM-IV and DSM-5 criteria. Journal of traumatic stress. 2013;26(5):537-47.

20. Organization for Economic Cooperation Development Staff (OECD). Health at a Glance 2013: OECD Indicators: OECD; 2013.

21. USA Social Security Administration. Actuarial Life Table 2010 [cited 2015 7/11/2015]. Available from: http://www.ssa.gov/oact/STATS/table4c6.html.

22. Actuarial Life Table: Official Social Security Website; 2015 [cited 2015]. Available from: http://www.ssa.gov/oact/STATS/table4c6.html.

23. Waldman P. American War Dead, By the Numbers. 2015.

24. Committee on the Assessment of the Readjustment Needs of Military Personnel V, and Their Families,, Base on Health of Select Populations, Institute of Medicine. Returning Home from Iraq and

Afghanistan: Assessment of Readjustment Needs of Veterans, Service Members, and Their Families. National Academies Press2013.
25. Homer JB. Partial-model testing as a validation tool for system dynamics (1983). System Dynamics Review. 2012;28(3):281-94.
