# Supplementary material for: A Dynamic Model of Post-Traumatic Stress Disorder for Military Personnel and Veterans
Source: PLoS One. 2016 Oct 7;11(10):e0161405. doi: 10.1371/journal.pone.0161405 (PMC5055362; doi:10.1371/journal.pone.0161405)
Supplement: S2 File — This zipped file includes the simulation model (PTSD_Simulation.mdl), data file (Data.vdf), as well as a ReadMe document (ReadMe.docx). See the ReadMe document for instructions on how to use the model and data. The simulation can also be run online without any software requirements at http://jalali.mit.edu/ptsd-simulation—PTSD_Simulation.mdl file includes more features; the online version is developed in a more interactive environment. (ZIP) [file pone.0161405.s002.zip › ReadMe.docx]

**INSTRUCTIONS TO WORK WITH THE SIMULATION MODEL**

**Setup:**

In order to run the simulation, you need to install Vensim. Vensim is available for Windows and Mac OS X and its version PLE can be downloaded for free at <http://vensim.com>. The simulation model can be run with any versions of Vensim.

**Note 1**: Before running the simulation, make sure the Vensim file and data are all in the same directory (folder). These files are named:

- *PTSD_Simulation.mdl*
- *Data.vdf*

**Simulation Dashboard:**

- Open *PTSD_Simulation.mdl* and make sure that you see the Dashboard view.
  - On the bottom left corner, you should see ‘Dashboard’ (see figure below). If you see something else, simply click on it and select Dashboard.


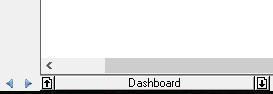


Figure 1: View selection

Let’s start with running the model:

1. **Run the simulation**: Run the model in SyntheSim mode by clicking on the icon shown below. The SyntheSim mode shows the results of the simulation superimposed on the chart to help you analyze the model outputs.


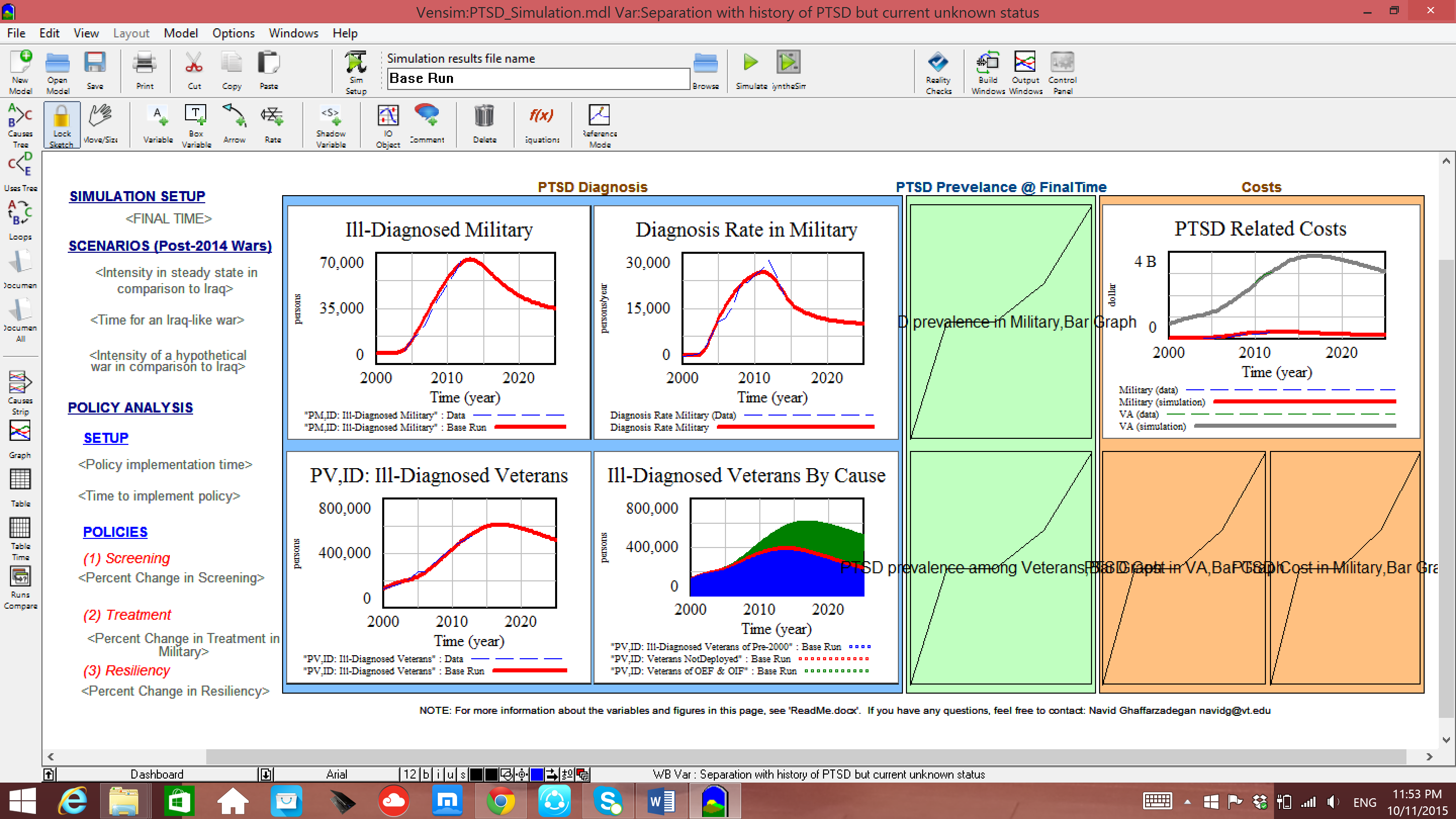


*Click to start the simulation in SyntheSim model*

Figure 2: Run the simulation in SyntheSim model

1. **Modify model parameters:** Once you click on the SyntheSim icon, each parameter (on the left side of the screen) will have a slider to be changed. You can simply move the slider under each parameter and Vensim automatically re-simulates the model and updates the figures.

Your screen should look like the following Figure.


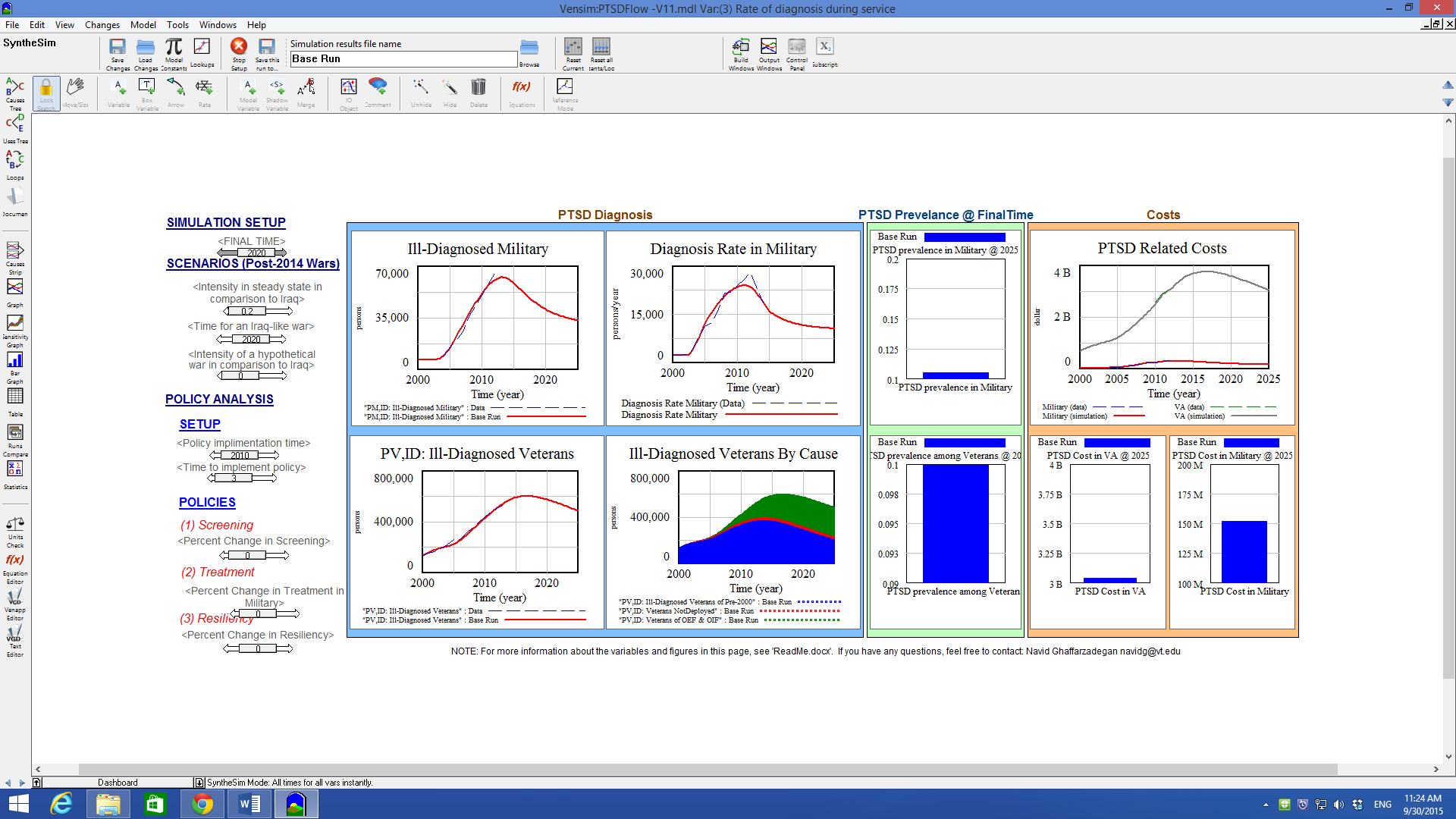
Figure 3: Dashboard view, after clicking on the icon ‘SyntheSim’

AS shown in the figure above, the Dashboard view includes several parameters (left side) and nine figures. The parameters help represent different scenarios and policies in the model, explained in details in the next section. The figures include PTSD diagnosis trends (Ill-diagnosed military (top-left), Diagnosis rate in military, Ill-diagnosed Veterans (bottom-left), and Ill-diagnosed veterans by Cause. The latter shows three sub-populations of veterans who have PTSD (pre-2000, veterans not-deployed, and veterans of OEF and OIF). Since some portion of PTSD patients at each time period are undiagnosed, we also report PTSD prevalence in the military and among veterans at the final time of the simulation (two bar-charts, around the middle of the screen). The last three graphs present cost-related variables: PTSD Related Costs (time series), PTSD cost in VA at the final simulation time, and PTSD cost in Military at the final simulation time.

1. **Reset your changes in the SyntheSim model**: After clicking on SyntheSim, the upper menu bar in Vensim changes and new buttons shown in Figure 4 appear, where you can reset the last modified slider to its value when SyntheSim was started or reset all sliders.

*Click on this button to reset the last modified slider to its value when the simulation started.*

*Click on this button to reset all sliders.*


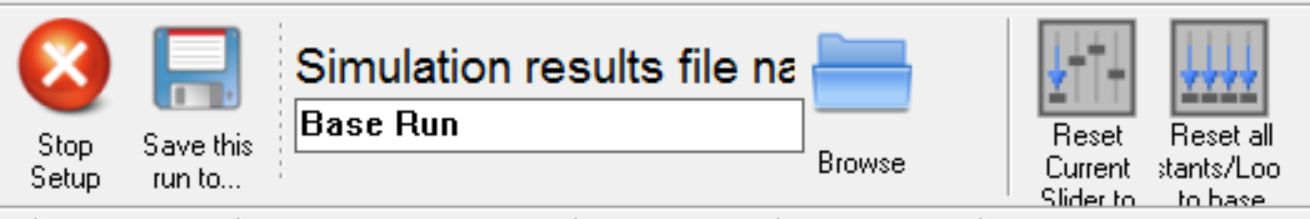


Figure 4: Buttons shown in the SyntheSim model

1. **Stop the simulation**: Simply click on ‘Stop Setup’ (Figure 4) to stop the SyntheSim mode.

**How to work with the parameters:**

- - SIMULATION SETUP
    - Final time: This parameter defines the last year of the simulation run. The default is 2025. This can be increased for policies that may have longer effects.
  - SCENARIOS (Post-2014 Wars):

Scenarios are environmental changes out of the scope of the healthcare system. In this model, the scenarios are about future involvement in wars. You can use parameters below to represent future scenarios about the US involvement in wars.

- - - Intensity in steady state in comparison to Iraq: in long runs, the US may keep some level of military personnel in vulnerable areas or may engage in more wars. Currently, this parameter is set to 0.1, which means that the number of people deployed to vulnerable areas are about 0.1 * the number of people deployed to Iraq at the time of the war. This will result in the total of 1% of military personnel serving in such areas, the policy supported by President Obama.

As a simulation test, change this parameter to 0.5. Please check simulation results as they change when you move the slider from 0.1 to 0.5. The change will represent a condition where 5% of the military personnel are deployed to areas like combat zones during the next decade.

- - - Time for an Iraq-like war & Intensity of a hypothetical war in comparison to Iraq:

These two parameters help simulate the effects of a war that may happen in the future with specific start and end times (such a war cannot be represented by a steady state deployment using the first parameter). As a test, set the ‘Time for an Iraq-like war’ to be 2017, and the ‘Intensity of a hypothetical war in comparison to Iraq’ to be 1, and check out the results. This will represent the effects of a war that happens in the same scale as the past war in Iraq, but starting in 2017.

- - POLICY ANALYSIS

Policy interventions are action that the healthcare system can do to mitigate the problems of PTSD.

- - SETUP
    - Policy implementation time: the time to implement a policy (see below for the policy choices). For example, if you select 2015, the policy will start at year 2015 (the current default in the model).
    - Time to implement policy: Once a policy is implemented, it is not effective immediately. Hence, there’s a delay after the implementation of the policy. This parameter (unit: year) helps you adjust such delay. Note that this number should be a nonzero positive number (the default value is 3 years).
  - POLICIES
    - Screening: This parameter defines the screening sensitivity. To change the screening sensitivity, change the parameter ‘Percent Change in Screening’. This parameter can be any number larger than -100. For instance, if it is defined to be -100, the baseline screening (in place up to the year of policy implementation) will be fully eliminated. If it is 200, it means that the screening is improved by 200%—twice more cases are diagnosed with PTSD.
    - Treatment: This policy allows you to adjust the effectivity of treatment. To change this effectiveness level, modify the parameter ‘Percent Change in Treatment in Military’. This parameter can be any number larger than -100. For example, if it is defined to be 300, the effectivity of treatment is improved by 300%.
    - Resiliency: To change the resilience of military personnel to PTSD, change the parameter ‘Percent Change in Resiliency’. This parameter can be any number between -100 and +100. For example, if you set it to be -50, the resiliency level will be reduced by half (50% reduction).

**Other views:**

In addition to the Dashboard view, there are several other views which present different sub-sections of the model. These views are useful for exploring the structure of the system, and how different variables are defined. As a model user you do not need to explore these views (but your welcome to do that!), and you can stay in the Dashboard view.

1. Healthy: This the substructure which keeps track of military personnel and veterans who do not have PTSD. The population of healthy people exit from this view as they get PTSD, or as they die.
2. PTSD OIF & OEF: This is the substructure which tracks PTSD in Iraq and Afghanistan wars and potential wars post-2014.
3. PTSD Pre-2000: This is the substructure which tracks PTSD cases from pre-2000 wars, such as the ones in Vietnam.
4. PTSD Non-combat related: This the substructure which tracks non-combat related PTSD incidents.
5. PTSD ALL: In this view, all the simulation runs from the previous views are used to calculate the total population of PTSD patients and PTSD prevalence.
6. Treatment: This is the view where you can track different scenarios about what may happen after people stop treatment (such that no one or all have been permanently cured). Since there is no data on the effectiveness level of current treatment procedures, this helps analyze the sensitivity of the outcomes to changes in the percentage of patients who get permanently cured vs. ones who will need to return to the healthcare system.
7. Policy tests: This is a view which operationalizes scenarios and policy tests.

If you have any questions about the model, please feel free to contact the corresponding author, [jalali@mit.edu](mailto:jalali@mit.edu).
